# Colchicine in Cardiac Surgery: The COCS Randomized Clinical Trial

**DOI:** 10.3390/jcdd9100363

**Published:** 2022-10-20

**Authors:** Vladimir Shvartz, Tatyana Le, Soslan Enginoev, Maria Sokolskaya, Artak Ispiryan, Elena Shvartz, Daria Nudel, Naylyana Araslanova, Andrey Petrosyan, Sergey Donakanyan, Igor Chernov, Leo Bockeria, Elena Golukhova

**Affiliations:** 1 Bakulev Scientific Center for Cardiovascular Surgery, 121552 Moscow, Russia; 2 Federal Center for Cardiovascular Surgery of the Russian Federation Ministry of Healthcare (Astrakhan), 414011 Astrakhan, Russia; 3 Astrakhan State Medical University of the Russian Federation Ministry of Healthcare, 414000 Astrakhan, Russia; 4 National Medical Research Center for Therapy and Preventive Medicine, 101990 Moscow, Russia

**Keywords:** colchicine, postoperative atrial fibrillation, coronary artery bypass grafting, aortic valve replacement

## Abstract

Background. In patients who underwent cardiac surgery, first-time postoperative atrial fibrillation (POAF) is associated with increased incidence of hospital-acquired complications and mortality. Systemic inflammation is one of confirmed triggers of its development. The anti-inflammatory properties of colchicine can be effective for the POAF prevention. However, the results of several studies were questionable and required further investigation. Hence, we aimed to evaluate the effectiveness of low-dose short-term colchicine administration for POAF prevention in patients after the open-heart surgery. This double-blind randomized placebo-controlled trial included 267 patients, but 27 of them dropped out in the course of the study. Study subjects received the test drug on the day before the surgery and on postoperative days 2, 3, 4 and 5. The rhythm control was conducted immediately after the operation and until the discharge from the hospital. The final analysis included 240 study subjects: 113 in the colchicine group and 127 in the placebo group. POAF was observed in 21 (18.6%) patients of the colchicine group vs. 39 (30.7%) control patients (OR 0.515; 95% Cl 0.281–0.943; *p* = 0.029). We observed no statistically significant differences between the patient groups in the secondary endpoints of the study (hospital mortality, respiratory failure, stroke, bleeding, etc.). For other parameters characterizing the severity of inflammation (pericardial effusion, pleural effusion, WBC count, neutrophil count), there were statistically significant differences between the groups in the early postoperative period (days 3 and 5). Also, statistically significant differences between the groups in the frequency of adverse events were revealed: the incidence of diarrhea in the colchicine group was 25.7% vs. 11.8% in the placebo group (OR 2.578; 95% Cl 1.300–5.111; *p* = 0.005); for abdominal pain, incidence values were 7% vs. 1.6%, correspondingly (OR 4.762; 95% Cl 1.010–22.91; *p* = 0.028). Thus, there were statistically significant differences between groups in the primary endpoint, thereby confirming the effectiveness of short-term colchicine use for the POAF prevention after coronary artery bypass grafting and/or aortic valve replacement. Also, we detected statistically significant differences between groups in the frequency of side effects to colchicine: diarrhea and abdominal pain were more common in the colchicine group. This clinical trial is registered with ClinicalTrials database under a unique identifier: NCT04224545.

## 1. Introduction

Postoperative atrial fibrillation (POAF) is a common complication in cardiac surgery occurring with a frequency of 15% to 50% [1,2]. It was established that POAF is associated with an increase in the incidence of early complications and mortality, along with the length of hospital stay and economic costs of patient treatment [3,4].

Systemic inflammation is among confirmed triggers of the atrial fibrillation (AF) development after a cardiac surgery [5,6,7], especially in patients with a cardiac electrophysiological substrate. Also, systemic inflammation contributes to the development of fibrosis in the myocardium of the left atrium and disruption of existing sympathetic/parasympathetic balance of autonomic control of the heart, etc. [8,9,10].

It was established that incidence of POAF increases with age, which is explained by the growing severity of fibrous changes in the left atrium. A degree of atrial tissue fibrosis is the most significant characteristic of atrial remodeling [1,2,3,4,5,6,7,8,9,10,11,12,13,14,15]. Prior paroxysmal AF several times increases the risk of developing POAF, since the pathophysiological mechanisms of arrhythmogenic cardiomyopathy result in a pronounced proarrhythmogenic condition [16]. 

In addition, the history of arrhythmia proved its effect on autonomic regulation of the entire cardiovascular system: an increase in the activity of the sympathetic division of the autonomic nervous system leads to a reduction in the duration of the action potential, whereas an imbalance in the parasympathetic division changes atrial refractoriness by increasing the influx of intracellular Ca^2+^ carriers [17,18]. 

The use of anti-inflammatory drugs in some studies had a positive effect on the incidence of POAF, confirming the role of systemic inflammation in its pathogenesis [19,20]. Colchicine can be effective for the prevention of POAF due to its anti-inflammatory properties. Several studies assessed the effect of colchicine on the incidence of POAF after open-heart surgery, but their results were equivocal [21,22,23,24,25,26,27]. In addition, meta-analyses and systematic reviews were conducted to combine the data of such studies [28,29]. The conclusions of the authors of the latest meta-analysis confirmed the potential effectiveness of colchicine, and 2014 AHA/ACC/HRS recommendations noted that postoperative colchicine administration may reduce AF in patients after cardiac surgery (Class IIb, Level of Evidence: B) [30]. However, in the latest ESC and AHA/ACC/HRS clinical guidelines, colchicine intake is not explicitly regulated: these guidelines state that the medicine is under investigation regarding its role in the prevention of POAF [31,32].

Besides, an important aspect to consider is the presence of gastrointestinal adverse reactions (nausea, vomiting, diarrhea, abdominal pain and lack of appetite) to colchicine intake demonstrated in most studies.

Hence, additional research in this field is required to find optimal doses and intake frequency of colchicine to minimize the side effects, while preserving its anti-inflammatory action.

Our study aimed to evaluate the effectiveness of low doses of short-term colchicine intake in the prevention of POAF in patients after open-heart surgery.

## 2. Materials and Methods

### 2.1. Study Design

Our study was a double-blind randomized placebo-controlled clinical trial. It was named “COlchicine in Cardiac Surgery” (COCS), registered at http://clinicaltrials.gov (with the latest access on 1 September 2022), and had the unique identifier of NCT04224545.

The research was performed at two Russian Federation Ministry of Healthcare institutions: Bakulev Center for Cardiovascular Surgery (Moscow, Russia) and Federal Center for Cardiovascular Surgery (Astrakhan, Russia). This study protocol complied with ethical guidelines of 1975 Declaration of Helsinki and with the Ethical Guidelines for Epidemiological Research by the Government of the Russian Federation. 

The study was approved by the human subjects review committees of all participating institutions and carried out in accordance with the international standards of good clinical practice. Written informed consent was obtained from each patient prior to randomization.

The design of our study was simple and pragmatic in order to maximize its likely practical application that could be extended to all patients. The data were collected by all authors and then verified and analyzed at the Bakulev Center for Cardiovascular Surgery after blinding the events.

### 2.2. Inclusion Criteria

Our study included patients 40–80 years of age scheduled for coronary artery bypass grafting (CABG) and/or aortic valve replacement (AVR).

### 2.3. Exclusion Criteria

The exclusion criteria were as follows: any form of AF, atrial flutter or supraventricular arrhythmias in the anamnesis, frequent ventricular or supraventricular extrasystole, AV blocks of 2nd and 3rd degrees, intake of steroids or any antiarrhythmic drugs except beta-blockers during the last month before surgery, previous open heart and chest surgeries, moderate to severe chronic renal insufficiency (creatinine clearance of <50 mL/min), chronic liver disease, mitral valve disease (insufficiency and/or stenosis of the grade > 2), and the patient’s participation in another clinical trial.

All of the above cardiac arrhythmias were not recorded in the patient prior to the inclusion in the study, according to ECG and Holter monitoring, which were carried out during the preoperative outpatient examination, as well as on the basis of complaints and anamnesis. 

The reasons for exclusion from the study after randomization were: hospital death on the postoperative day 1; intensive care unit (ICU) stay after surgery delayed by more than 1 day, which prevented the patient from receiving the study drug; and the patient’s wish to withdraw from the study.

### 2.4. Randomization

The patients were randomly distributed between two groups: the experimental group received colchicine at the dose of 1 mg once a day, while the control group received placebo according to the same scheme. COLCHICINA LIRCA^®^ 1 mg (ACARPIA Farmaceutici Srl., Milan, Italy) was used in this study. The schedule for administration was as follows: 24 h before surgery and on postoperative days 2, 3, 4 and 5, in combination with optimally selected medicamentous therapy. Random allocation to treatment groups was carried out using a centralized computer-based automated sequence. Randomization was based on permuted blocks with a block size of 20. The randomization sequence was implemented using sequentially numbered study drug containers. Allocation concealment was achieved through the use of opaque sealed envelopes, sequentially numbered containers, and centralized randomization.

### 2.5. Sample Size Calculation

Previously published studies had a clear heterogeneity of patients in terms of factors associated with an increased risk of POAF, as well as heterogeneity in the types and volumes of surgical interventions. Consequently, the data were scattered on a larger scale regarding incidence of POAF in different studies. Therefore, to assess the variance in POAF rates, we performed a preliminary analysis of our data after randomization of 100 patients. According to the inclusion/exclusion criteria and selected surgical interventions (CABG and/or AVR), at the preliminary stage of the analysis, it was shown that incidence rates of POAF were 18% in the experimental group vs. 29.4% in the control group [33].

Using the formula proposed by R. Lehr [34], taking into account the data on the incidence of the studied event (POAF) in our sample of patients, the estimated number of observations should have been at least 223 with a given statistical power of 80% and α=0.05. Accordingly, we planned to establish a representative sample size in the final analysis of at least 230 patients.

### 2.6. Statistical Analyses

Statistical analysis was carried out using STATISTICA^®^ (Statsoft, Palo Alto, CA, USA) and SPSS^®^ Statistics 25.0 software (IBM, Armonk, NY, USA). The data are presented in the form of median and interquartile range—Me (Q1; Q3), and frequencies. To compare two independent samples, we used Mann–Whitney U test for quantitative variables, and Pearson’s chi-squared test or Fisher’s exact test for categorical variables. The difference between the groups was assumed statistically significant at *p* < 0.05.

### 2.7. Endpoints: Primary Outcome Measure

Number of study subjects with POAF. POAF was detected by continuous ECG monitoring carried out immediately after the operation and continued until the end of postoperative day 7. Diagnostic confirmation of POAF was an episode with the absence of visible regular P waves and appearance of F waves and irregular RR intervals on the ECG for more than 30 s.

### 2.8. Endpoints: Secondary Outcome Measure

Number of study subjects with fatal and non-fatal events. The main nosocomial non-fatal events are stroke, bleeding, respiratory failure, infectious complications, etc. Pericardial effusion and pleural effusion were assessed via echocardiography on postoperative days 3 and 5. The dynamics of inflammation biomarkers and biomarkers of liver damage (aspartate aminotransferase, alanine aminotransferase) in blood plasma (leukocytes, neutrophils) was evaluated the day before the operation, as well as on postoperative days 3 and 5. 

### 2.9. Surgery

CABG was performed on a beating heart with cardiopulmonary bypass (parallel perfusion) or in off-pump mode, depending on the preferences of the operating surgeon. Conventionally, the left internal thoracic artery was used as a conduit with bypass of the anterior interventricular artery, while great saphenous vein was used to bypass the basins of the remaining coronary arteries. Occasionally, the radial artery was used. In cases of combined CABG with AVR, the first stage was the collection of conduits in the planned number, followed by performing the AVR. After restoring the integrity of the aorta and the right atrium, the patient’s body was warmed up to 36.6 °C, and cardiac activity was restored. Next, we performed myocardial revascularization on a beating heart under cardiopulmonary bypass. The quality of the formed anastomoses was assessed via intraoperative shuntography, thereby allowing intraoperative detection and elimination of anastomotic leakages.

### 2.10. Monitoring

All patients underwent continuous ECG monitoring: 3-channel ECG monitoring in the ICU on postoperative days 1 and 2, 10-min 12-lead ECG recording daily from the day 3 until discharge from the hospital, as well as at any time when a patient complained of a heartbeat. On postoperative days 3 and 5, 24-h Holter monitoring was performed. Also, all participants underwent transthoracic echocardiography and laboratory blood tests on postoperative days 3 and 5. The development of POAF was defined as an episode with the absence of visible regular P waves, appearance of F waves and irregular RR intervals on the ECG for more than 30 s. On day 7, the patient was asked about the presence of any of the listed adverse events (nausea, diarrhea, etc.) in the postoperative period.

## 3. Results

**Figure 1:**
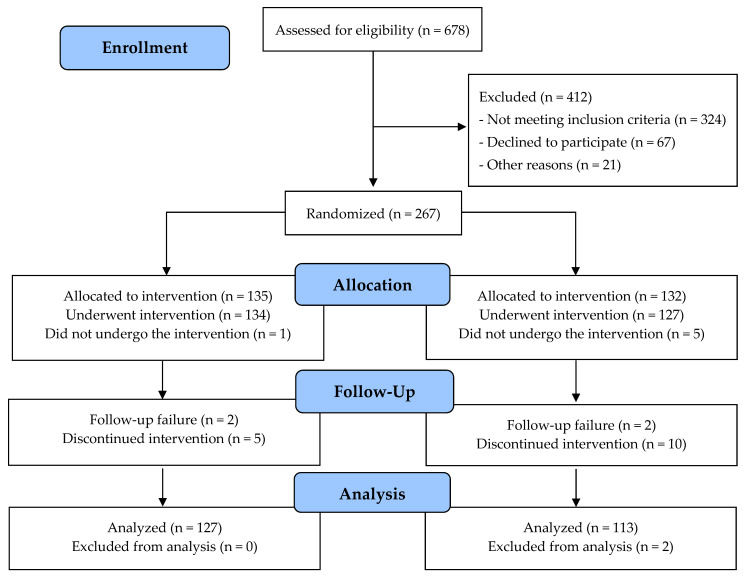
CONSORT flow diagram.

A total of 267 patients were randomized, of which 27 subjects dropped out of the study: 19 from the experimental group and eight from the control group (Figure 1). The reasons for exclusion from the experimental group were: prolonged stay in the ICU (n = 10), change in the treatment protocol (n = 5), death on postoperative day 1 (n = 2), and incomplete instrumental examinations (n = 2). The reasons for exclusion from the control group were: prolonged stay in the ICU (n = 5), death on postoperative day 1 (n = 1), change in the treatment protocol (n = 1), and willingness to withdraw from the study (n = 1).

Consequently, 240 patients were included in our analysis: 113 from the experimental group and 127 from the control group. Initial clinical, laboratory and instrumental data, as well as medicamentous therapy and intraoperative data did not differ statistically significantly between the groups (Table 1 and Table 2), with the exception of cardiotonic support frequency in the ICU, which was higher in the experimental group (*p* = 0.010).

POAF was observed in 21 (18.6%) subjects in the experimental group vs. 39 (30.7%) subjects in the control group. This difference was statistically significant (OR 0.515; 95% Cl 0.281–0.943; *p* = 0.029) (Table 3).

**Figure 2:**
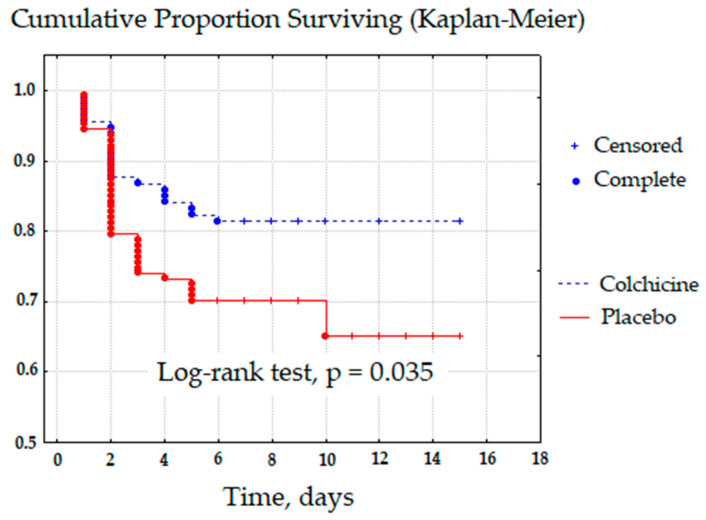
Kaplan-Meier survival and absence of POAF.

When comparing patient groups for survival by Kaplan–Meier and the absence of POAF, we obtained a statistically significant log-rank test (*p* = 0.035) (Figure 2). However, we detected no statistically significant differences in secondary endpoints of the study (hospital mortality, respiratory failure, stroke, bleeding, etc.) (Table 3).

AV repair and colchicine use were statistically significant parameters in the overall cohort of patients when building a multivariate Cox regression model (Table 4). However, based on some parameters characterizing the severity of inflammation (pericardial effusion, pleural effusion, WBC count, neutrophil count), there were differences between the groups in the early postoperative period on days 3 and 5 (Table 5 and Table 6).

There were no statistically significant differences between the groups on postoperative days 3 and 5 in terms of laboratory test results regarding liver and kidney functioning (Table 6). Medicamentous therapy in the postoperative period did not differ as well. There were statistically significant differences in adverse events between groups. The incidence of diarrhea in the colchicine group was 25.7% vs. 11.8% in the placebo group (OR 2.578; 95% CI 1.300–5.111; *p* = 0.005). The incidence of abdominal pain in the colchicine group was 7% compared with 1.6% in the placebo group (OR 4.762; 95% CI 1.010–22.91; *p* = 0.028) (Table 7).

## 4. Discussion

In this study, we investigated the efficacy of short-term low-dose colchicine administration for the prevention of newly developed POAF after CABG and/or AVR. We have obtained convincing data on its effectiveness: in the colchicine group, POAF was observed in 18.6% of patients vs. 30.7% in the placebo group (OR 0.515; 95% CI 0.281–0.943; *p* = 0.029).

Since POAF most often develops during the first 2-4 days after surgery, preoperative administration of colchicine is crucial. In our study, all patients received colchicine once on the day preceding surgery, as well as during four postoperative days. In the COPPS POAF study, patients received the studied drug starting from postoperative day 3; and the results were evaluated exclusively thenceforth [21]. Thus, the COPPS POAF study did not evaluate postoperative days 1 and 2, when the likelihood of developing POAF was highest.

The pronounced anti-inflammatory effect was directly related to the high dose of colchicine, as well as the severity of gastrointestinal side effects. Different studies used different schedules and doses of colchicine intake. In the study [21], colchicine was administered at a dose of 1.0 mg twice on the first day, followed by a maintenance dose of 0.5 mg twice daily for 1 month in patients weighing over 70 kg. In patients weighing less than 70 kg or with intolerance to the highest dose, half doses were administered. The authors observed the anti-inflammatory efficacy of colchicine, but a high incidence of gastrointestinal adverse events was noted as well. In the study [27], the authors used the following regimen: patients received 1 mg of colchicine or an appropriate placebo 12–24 h before surgery, then colchicine (0.5 mg) or placebo immediately after surgery, followed by daily treatment at this dose until discharge from the hospital. In that study, colchicine was well tolerated, but there was no statistically significant effect on the primary endpoint.

In our study, patients received 1 mg of colchicine per day; that is, in total, each patient received 5 mg over 6 days. Such low dose made it possible to reduce the severity of adverse gastrointestinal events and preserve anti-inflammatory effect of colchicine. It is important to reduce the dose to minimize the severity of side effects, as these side effects constitute the reason discontinuing colchicine intake. Unfortunately, side effects in our study were still significantly more common in the colchicine group, with an incidence of diarrhea of 25.7% vs. 11.8% in the placebo group, and an incidence of abdominal pain of 7% vs. 1.6%, correspondingly. However, the severity of side effects was not statistically significant, as only one patient in the control group wished to terminate participation in the study prematurely.

In order to reduce the effect of other important and already confirmed in multiple studies risk factors for the development of POAF, which could affect the integrity of the experiment, we defined exclusion criteria for this study. In our opinion, it was important to exclude patients with any previous form of atrial fibrillation/flutter and any history of supraventricular arrhythmias. We also excluded patients with mitral valve disease, often accompanied by AF. In addition, we did not include patients with previously performed heart and chest surgeries, since existing adhesions and scar structures may be a substrate for the development of AF. Thus, we initially excluded patients at high risk of developing AF in order to minimize the effect of existing predictors of AF occurrence in the preoperative period.

Also, we performed just two types of heart surgery: CABG and AVR, which anatomically and pathophysiologically were not associated with either the left atrium or the pulmonary veins, the involvement of which in surgical procedures during surgical interventions could be a trigger for an increase in the risk of POAF. In our opinion, this issue could take place in previous studies with colchicine: for example, in the COPPS-2 study [22], as well as in studies [25] and [27], where the authors did not find a statistically significant benefit of taking colchicine, and surgical operations with interventions on the mitral valve were performed in the patients. The incidence of POAF in both studies tended to differ between groups, but with such low variance value, the patient sample size was probably insufficient.

The study by Sarzaeem et al. [23] included solely patients after CABG (n = 216). The authors observed statistically significant difference in the incidence of POAF in the colchicine group (14.8%) vs. the control group (30.6%) (*p* = 0.006). ICU stay in the colchicine group was 2.4 ± 1.3 days vs. 3.1 ± 1.5 days in the control group (*p* < 0.001), and hospital stay in the colchicine group was 6.6 ± 1.5 days vs. 8.1 ± 2.0 days in the control group (*p* < 0.001).

In another study [26], where the authors examined the effectiveness of colchicine in relation to the development of postcardiotomy syndrome, an additional primary endpoint was the incidence of POAF. Although the authors reported enrollment of 240 patients (2 groups, 120 in each), only 29 patients in the colchicine group and 52 patients in the placebo group were reported in the article tables. Unfortunately, it is difficult to draw reliable conclusions from this publication, given its inconsistency. This article was also cited in meta-analyses as inconsistent data in studies involving colchicine.

For each specific combination of the somatic features of the patient (history of paroxysmal AF, mitral valve disease, chronic inflammatory diseases, initial antiarrhythmic drug therapy, etc.), and for each specific volume of surgical intervention (isolated CABG, CABG combined with AVR or with mitral valve replacement, intervention on the ascending aorta, etc.), there is a specific effectiveness of colchicine, and the increase of this effectiveness would require the presence of statistically significant associations in the sample of a certain size. In our study, we first obtained pilot data on the frequency of POAF in our cohort, and then calculated the required minimum sample size, which was necessary for a given frequency of the phenomenon under study in our population. 

Hence, in our opinion, the anti-inflammatory properties of colchicine can be used to reduce inflammation after heart surgery: the effectiveness of colchicine in the prevention of POAF is convincing. Conflicting data were obtained in studies with a high degree of heterogeneity in the parameters involved in the POAF development. To obtain statistically significant differences, it is necessary either to reduce such heterogeneity, as we did in our study, or to increase the statistical power of the study by means of a larger sample size. 

However, reducing the dose and frequency of colchicine intake could not result in the absence of gastrointestinal adverse events in the patients of our study. It is likely that the positive effect of colchicine in the prevention of POAF correlates with its negative side effects. It is impossible to entirely avoid the side effects while trying to preserve the anti-inflammatory properties of colchicine. In this situation, physicians may need to make an individual choice in case of each individual patient regarding the expediency of taking colchicine for the risk reduction of developing POAF.

In fact, our study protocol differed substantially from previous studies in terms of more homogeneous patient groups and the absence of high-risk factors for the AF development. Our results should be used for future meta-analyses and for calculation of the required number of patients in the course of planning larger randomized trials.

### Limitations of the Study

To assess inflammation in the postoperative period, we used the levels of leukocytes and neutrophils in the blood as inflammation markers, however, markers of the interleukin family (IL-1, IL-6, IL-10), TNFa, NLRP3, etc., are more specific.

Another limitation of our study was that asymptomatic episodes of POAF could have been partially missed with this type of rhythm control. However, the likelihood of their development was similar in both groups in a randomized, blinded study; hence, we believe that this particular limitation did not have a significant effect on the results of our research.

## 5. Conclusions

We obtained convincing evidence of the efficacy of short-term colchicine intake in the prevention of POAF after CABG and/or AVR. In the experimental group, POAF was observed in 21 (18.6%) patients vs. 39 (30.7%) subjects in the control group. These differences were statistically significant (OR 0.515; 95% CI 0.281–0.943; *p* = 0.029).

There were also statistically significant differences between the groups in the frequency of adverse reactions to colchicine. The incidence of diarrhea in the experimental group was 25.7% vs.11.8% in the control group (OR 2.578; 95% CI 1.300–5.111; *p* = 0.005). The corresponding values for abdominal pain were 7% versus 1.6% (OR 4.762; 95% CI 1.010–22.91; *p* = 0.028).

## Figures and Tables

**Table 1:** Patient parameters according to the initial data.

Parameters	Colchicine (n = 113)	Placebo (n = 127)	*p*
Clinical parameters of patients			
Age, y	62 (55; 67)	61 (56; 67)	0.851
Male, n (%)	83 (73.5)	97 (76.4)	0.601
BSA, m^2^	2.01 (1.9; 2.18)	2.02 (1.89; 2.13)	0.806
Weight, kg	84 (74; 94)	85 (76; 94)	0.493
BMI, kg/m^2^	29 (26; 31.9)	29 (26; 32.3)	0.638
Angina pectoris, n (%)	103 (91)	112 (88)	0.454
Angina pectoris–classes III–IV, n (%)	59 (52)	67 (53)	0.837
Diabetes, n (%)	28 (24.7)	24 (19)	0.270
COPD, n (%)	7 (6.2)	7 (5.5)	0.822
Hypertension, n (%)	100 (88.5)	119 (93.7)	0.155
Previous AMI, n (%)	46 (40.7)	51 (40)	0.931
Stroke, n (%)	2 (1.7)	2 (1.6)	0.906
Smoking, n (%)	25 (22.1)	41 (32.3)	0.079
Echocardiographic parameters			
LVEF, %	60 (56; 64)	60 (56; 64)	0.507
iESD	16.5 (15.3; 18.1)	16.5 (15; 18)	0.644
iEDD	24.8 (22.8; 26.5)	24 (22.7; 26.2)	0.674
iESV	22.6 (18.7; 26.7)	21.5 (18.4; 26.1)	0.423
iEDV	56.3 (48.6; 66.7)	55.8 (46.8; 64.4)	0.562
MR, degree	1.5 (1; 1.5)	1.5 (1; 1.5)	0.492
AR, degree	1 (0; 1)	0 (0; 1)	0.577
IVS, mm	13 (12; 15)	13 (12; 15)	0.811
LA size, cm	4 (3.8; 4.4)	4 (3.7; 4.4)	0.964
iLA size, cm	2 (1.9; 2.17)	2 (1.8; 2.22)	0.782
Laboratory test results			
WBC, 10^×^9/L	7.5 (6.4; 8.9)	7.6 (6.6; 9.1)	0.514
Neutrophils, 10^×^9/L	4.4 (3.4; 5.4)	4.7 (3.7; 5.3)	0.619
Neutrophils, %	58 (53; 65)	58.9 (53.2; 63)	0.677
Platelets,10^×^9/L	236 (197; 279)	258 (213; 298)	0.197
Creatinine, mcmol/L	83.8 (73; 93.6)	84 (74; 95)	0.840
eGFR, mL/min	93.5 (79.8; 107.3)	89 (75.5; 110)	0.940
Glucose, mmol/L	5.6 (5; 6.4)	5.4 (4.9; 5.8)	0.101
AST, IU/L	22 (17; 27)	20 (17; 26)	0.520
ALT, IU/L	23 (16; 33)	24.5 (18; 34)	0.439
Potassium, mmol/L	4.4 (4.1; 4.8)	4.4 (4.1; 4.7)	0.990
Medicamentous therapy			
Beta-blockers, %	84 (74.3)	95 (74.8)	0.934
ACE inhibitors, %	69 (61)	72 (56.7)	0.493
Calcium antagonists, %	37 (32.7)	46 (36.2)	0.572
Thiazide diuretics, %	14 (12)	7 (5.5)	0.060
Loop diuretics, %	13 (11.5)	10 (7.9)	0.341
Potassium-sparing diuretics, %	24 (21)	22 (17.3)	0.442
NSAIDs, n (%)	(0)	0 (0)	
Acetylsalicylic acid, n (%)	36 (31.8)	44 (34.6)	0.648
Other antiaggregant, n (%)	22 (19.5)	22 (17.3)	0.668
Nitrates, %	29 (25.7)	25 (19.7)	0.269
Statins, %	77 (68)	101 (79.5)	***0.044***
LMWHs, n (%)	27 (23.9)	29 (22.8)	0.846

BSA—body surface area, BMI—body mass index, COPD—chronic obstructive pulmonary disease, AMI—acute myocardial infarction, LVEF—left ventricular ejection fraction, iESD—end-systolic dimension index, iEDD—end-diastolic dimension index, iESV—end-systolic volume index, iEDV—end-diastolic volume index, MR—mitral regurgitation, AR—aortic regurgitation, IVS—interventricular septum, LA—left atrium, iLA—index left atrium, WBC—white blood cell count, eGFR—estimated glomerular filtration rate, AST—aspartate aminotransferase, ALT—alanine aminotransferase, ACE—angiotensin-converting enzyme, NSAIDs—non-steroidal anti-inflammatory drugs, LMWHs—low-molecular-weight heparins.

**Table 2:** Operative and postoperative data.

Parameters	Colchicine (n = 113)	Placebo (n = 127)	*p*
CPB, n (%)	86 (76)	100 (78.7)	0.626
CPB time, min	105 (75; 130)	108 (80; 130)	0.533
Cardioplegia, n (%)	32 (28.3)	33 (26)	0.685
ACC time, min	62.5 (57; 66.5)	63 (54; 70)	0.928
CABG, n (%) AC–1 AC–2 AC–3 VC–1 VC–2 VC–3 VC–4	92 (81.4) 63 (55.7) 4 (3.5) 0 (0) 39 (34.5) 35 (30.9) 9 (7.9) 3 (2.6)	108 (85) 77 (60.6) 5 (3.9) 1 (0.8) 34 (26.7) 34 (26.7) 26 (20.5) 1 (0.8)	0.453
AV repair, n (%)	32 (28.3)	28 (22)	0.263
Cardiotonic support in ICU, n (%)	48 (42.5)	34 (26.7)	***0.010***
Lung ventilation time, h	8.3 (5.8; 13.6)	8.6 (5.8; 14.8)	0.750
Length of stay, days	7 (7; 8)	7 (6; 9)	0.679
**Subgroup CABG**			
**Parameters**	**Colchicine** **(n = 81)**	**Placebo** **(n = 99)**	***p***
CPB, n (%)	54 (66.7)	72 (72.7)	0.378
CPB time, min	83 (60; 106)	97 (75; 128)	***0.030***
Cardiotonic support in ICU, n (%)	27 (33.3)	20 (20.2)	***0.046***
Lung ventilation time, h	8 (5.9; 11.8)	8.2 (5.4; 12.3)	0.894
**Subgroup AV repair**			
**Parameters**	**Colchicine** **(n = 32)**	**Placebo** **(n = 28)**	***p***
CPB time, min	131 (113; 149)	123 (102; 135)	0.177
ACC time, min	62.5 (57; 66.5)	64.5 (54.5; 71.5)	0.899
AV repair + CABG, n (%)	11 (34.4)	9 (32)	0.854
Cardiotonic support in ICU, n (%)	21 (65.6)	14 (50)	0.221
Lung ventilation time, h	9.8 (5.7; 17.4)	12 (8; 17.3)	0.528

CPB—cardiopulmonary bypass, ACC—aortic cross-clamp, CABG—coronary artery bypass grafting, AC—arterial conduits, VC—venous conduits, AV—aortic valve, ICU—intensive care unit.

**Table 3:** Clinical outcomes and complications.

Parameters	Colchicine (n = 113)	Placebo (n = 127)	OR	95% CI	*p*
POAF, n (%)	21 (18.6)	39 (30.7)	0.515	0.281–0.943	***0.029***
Hospital mortality, n (%)	0 (0)	0 (0)			
Respiratory failure, n (%)	0 (0)	0 (0)			
Stroke, n (%)	0 (0)	0 (0)			
Bleeding, n (%)	0 (0)	0 (0)			
Pericardial puncture, n (%)	(0)	0 (0.8)			
Infectious complications of postoperative wound, n (%)	0 (0)	0 (0)			
Arrhythmias, except AF, n (%)	(3.5)	(3.2)	1.128	0.275–4.621	0.866
SVES, n (%)	(0.9)	(3.2)	0.274	0.031–2.493	0.202
Pacemaker implantation, n (%)	(2.6)	0 (0)			
**Subgroup CABG**					
POAF	8 (12)	25 (25.3)	0.417	0.187–0.930	***0.026***
Arrhythmias, except AF, n (%)	1 (1.2)	1 (1)	1.225	0.075–19.89	0.887
SVES, n (%)	1 (1.2)	3 (3)	0.400	0.041–3.921	0.402
**Subgroup AV repair**					
POAF	11 (34.4)	14 (50)	0.524	0.185–1.481	0.220
Arrhythmias, except AF, n (%)	3 (9.4)	3 (10.7)	0.862	0.159–4.659	0.863
SVES, n (%)	0 (0)	1 (3.6)			

POAF—postoperative atrial fibrillation, AF—atrial fibrillation, SVES—supraventricular extrasystole, OR—odds ratio, CI—confidence interval.

**Table 4:** Parameterization of Cox regression model for assessing the risk of developing POAF (χ^2^ = 17.3; *p* = 0.0083).

Parameters	Beta	Standard Error	*t*-Value	Exponent Beta	Wald Statistic	*p*
AV repair	0.808	0.306	2.637	2.244	6.954	***0.008***
Colchicine use	−0.567	0.274	−2.068	0.567	4.278	***0.038***
Cardiotonic support in ICU	−0.273	0.292	−0.934	0.760	0.872	0.350
Hypertension	0.461	0.601	0.766	1.586	0.587	0.443
Previous AMI	−0.231	0.325	−0.711	0.793	0.508	0.476
Male	−0.182	0.290	−0.626	0.833	0.392	0.531

AV—aortic valve, ICU—intensive care unit, AMI—acute myocardial infarction.

**Table 5:** Postoperative echocardiographic parameters.

Parameters	Colchicine (n = 113)	Placebo (n = 127)	*p*
Postoperative day 3			
LVEF, %	55 (52; 56.8)	55 (53; 57)	0.182
iESV	19.8 (16.2; 25.2)	20.1 (17.3; 23.8)	0.954
iEDV	44.4 (40; 54.4)	44.4 (39.4; 51.2)	0.372
Pericardial effusion, n (%)	11 (9.5)	20 (15.8)	0.146
Pericardial effusion, mm	3.5 (2.5; 4)	5 (5; 9)	***0.006***
Pleural effusion, n (%)	48 (43)	51 (40.5)	0.698
Pleural effusion, mm	18 (12; 20)	20 (15; 26)	0.063
Postoperative day 5			
LVEF, %	55.6 (53; 58)	55 (54; 58)	0.511
iESV	19.7 (16.2; 23.4)	19.6 (16.6; 23.8)	0.867
iEDV	45.3 (38.3; 53.3)	45 (38.5; 53.9)	0.818
Pericardial effusion, n (%)	23 (20.4)	28 (22.1)	0.745
Pericardial effusion, mm	5 (3; 6)	5 (5; 6)	0.122
Pleural effusion, n (%)	49 (43.3)	60 (47)	0.491
Pleural effusion, mm	19.5 (10; 26)	20 (15; 32)	***0.034***

LVEF—left ventricular ejection fraction, iESV—end-systolic volume index, iEDV—end-diastolic volume index.

**Table 6:** Postoperative laboratory test results.

Parameters	Colchicine (n = 113)	Placebo (n = 127)	*p*
Postoperative day 3			
WBC, 10^×^9/L	11.4 (9.5; 14.2)	12 (9.6; 14.6)	0.284
Neutrophils, 10^×^9/L	8.8 (6.7; 12)	8.9 (7.8; 12.7)	0.536
Neutrophils, %	77 (71; 84)	79 (72; 82)	0.901
Platelets,10^×^9/L	189 (160; 237)	199 (162; 254)	0.442
Creatinine, mcmol/L	72 (65; 82)	75 (66.7; 88.5)	0.065
eGFR	107 (90; 119)	100 (84; 124)	0.216
Glucose, mmol/L	7 (5.7; 8.6)	6.6 (5.7; 8.3)	0.441
AST, IU/L	31 (24; 45)	31 (21; 40)	0.494
ALT, IU/L	20 (14; 30)	21 (13; 29)	0.995
Potassium, mmol/L	4.1 (3.8; 4.3)	4.2 (3.9; 4.5)	0.056
Postoperative day 5			
WBC, 10^×^9/L	9.3 (8; 11)	10.9 (8.4; 13.2)	***0.003***
Neutrophils, 10^×^9/L	5.9 (4.7; 7.5)	6.8 (5; 9.4)	***0.014***
Neutrophils, %	62 (58.6; 68.9)	63 (57; 68)	0.982
Platelets, 10^×^9/L	265 (211; 314)	262 (217; 339)	0.631
Creatinine, mcmol/L	75 (68; 83)	77 (68; 86)	0.566
eGFR	103 (85; 119)	100 (82; 127)	0.755
Glucose, mmol/L	6.4 (5.3; 7.4)	5.9 (5.3; 7.3)	0.905
AST, IU/L	28 (22; 34)	29 (20; 38)	0.885
ALT, IU/L	28 (20; 42)	27 (17; 46)	0.886
Potassium, mmol/L	4.1 (3.7; 4.4)	4.3 (4; 4.6)	***0.011***

WBC—white blood cell count, eGFR—estimated glomerular filtration rate, AST—aspartate aminotransferase, ALT—alanine aminotransferase.

**Table 7:** Adverse clinical events.

Parameters	Colchicine (n = 113)	Placebo (n = 127)	OR	95% Cl	*p*
Nausea, n (%)	14 (12.4)	15 (11.8)	1.055	0.486–2.296	0.891
Vomiting, n (%)	2 (1.8)	6 (4.7)	0.364	0.072–1.839	0.191
Lack of appetite, n (%)	19 (16.9)	24 (18.9)	0.867	0.447–1.676	0.674
Diarrhea, n (%)	29 (25.7)	15 (11.8)	2.578	1.300–5.111	***0.005***
Abdominal pain, n (%)	8 (7)	2 (1.6)	4.762	1.010–22.91	***0.028***
Convulsions, n (%)	2 (1.8)	7 (5.5)	0.309	0.063–1.518	0.115
Tingling in hands and feet, n (%)	9 (8)	10 (7.8)	1.012	0.396–2.588	0.979
Skin rashes, n (%)	0 (0)	0 (0)			

OR—odds ratio, CI—confidence interval.

## Data Availability

The data sets analyzed in our study are publicly available in Supplementary Materials.

## Supplementary Materials

The following supporting information can be downloaded at: https://www.mdpi.com/article/10.3390/jcdd9100363/s1.
