# Supplementary material for: Colchicine in Cardiac Surgery: The COCS Randomized Clinical Trial
Source: J Cardiovasc Dev Dis. 2022 Oct 20;9(10):363. doi: 10.3390/jcdd9100363 (PMC9604685; doi:10.3390/jcdd9100363)
Supplement: Supplementary file 1 [file jcdd-09-00363-s001.zip › jcdd-1922103-supplementary.pdf]

All patients n=240								
	Mean	Median	Minimum	Maximum	Lower Quartile	Upper Quartile	Std. Dev.	Std. Error
Grouping	0,4708	0,0000	0,00000	1,000	0,0000	1,0000	0,5002	0,03229
Age	61,3333	62,0000	42,00000	80,000	55,0000	67,0000	8,3062	0,53616
Male	0,7500	1,0000	0,00000	1,000	0,5000	1,0000	0,4339	0,02801
BSA	2,0216	2,0200	1,53000	2,610	1,8900	2,1450	0,2000	0,01291
Weight	85,4667	85,0000	54,00000	129,000	75,0000	94,0000	14,6507	0,94570
BMI	29,3570	28,8700	20,00000	46,070	25,9850	32,0800	4,6032	0,29714
Angina pectoris	0,8958	1,0000	0,00000	1,000	1,0000	1,0000	0,3061	0,01976
Angina pectoris class III-IV	0,5292	1,0000	0,00000	1,000	0,0000	1,0000	0,5002	0,03229
Diabetes	0,2167	0,0000	0,00000	1,000	0,0000	0,0000	0,4128	0,02665
COPD	0,0583	0,0000	0,00000	1,000	0,0000	0,0000	0,2349	0,01516
Hypertension	0,9125	1,0000	0,00000	1,000	1,0000	1,0000	0,2832	0,01828
Prior AMI	0,4042	0,0000	0,00000	1,000	0,0000	1,0000	0,4918	0,03174
Stroke	0,0167	0,0000	0,00000	1,000	0,0000	0,0000	0,1283	0,00828
Smoking	0,2750	0,0000	0,00000	1,000	0,0000	1,0000	0,4474	0,02888
Beta-blockers	0,7458	1,0000	0,00000	1,000	0,0000	1,0000	0,4363	0,02816
ACE inhibitors	0,5875	1,0000	0,00000	1,000	0,0000	1,0000	0,4933	0,03184
Calcium antagonists	0,3458	0,0000	0,00000	1,000	0,0000	1,0000	0,4766	0,03077
Thiazide diuretics	0,0875	0,0000	0,00000	1,000	0,0000	0,0000	0,2832	0,01828
Loop diuretics	0,0958	0,0000	0,00000	1,000	0,0000	0,0000	0,2950	0,01904
Potassium-sparing diuretics	0,1917	0,0000	0,00000	1,000	0,0000	0,0000	0,3944	0,02546
NSAIDs	0,0000	0,0000	0,00000	0,000	0,0000	0,0000	0,0000	0,00000
Acetylsalicylic acid	0,3333	0,0000	0,00000	1,000	0,0000	1,0000	0,4724	0,03049
Other antiaggregant	0,1833	0,0000	0,00000	1,000	0,0000	0,0000	0,3877	0,02503
Nitrates	0,2250	0,0000	0,00000	1,000	0,0000	0,0000	0,4185	0,02701
Statins	0,7417	1,0000	0,00000	1,000	0,0000	1,0000	0,4386	0,02831
LMWHs/ UFH	0,2333	0,0000	0,00000	1,000	0,0000	0,0000	0,4238	0,02736
LVEF	59,4067	60,0000	36,20000	77,000	56,0000	64,0000	6,4697	0,41762
iESD	16,6430	16,4948	1,66667	28,800	15,0000	18,0328	3,0292	0,20854
iEDD	24,5745	24,4000	14,00000	35,000	22,7498	26,5097	3,2689	0,22451
iESV	23,8167	21,8000	7,10000	69,900	18,6164	26,5297	9,0084	0,58271
iEDV	58,0413	56,1000	25,80000	126,779	48,1125	65,4680	15,4347	0,99630
AV peak gradient	28,6625	8,0000	3,00000	143,000	6,0000	34,0000	37,1495	2,43898
AV mean gradient	20,7716	5,5000	0,00000	94,000	3,0000	42,0000	24,0311	1,88806
MR	1,3080	1,5000	0,00000	2,500	1,0000	1,5000	0,5289	0,03436
AR	0,7058	0,0000	0,00000	3,000	0,0000	1,0000	0,8451	0,05621
IVS	13,3136	13,0000	7,00000	22,000	12,0000	15,0000	2,5689	0,16722
LA size	4,5519	4,0000	2,90000	43,000	3,8000	4,4000	4,3793	0,28387
WBC	7,6757	7,5235	3,69000	12,200	6,4100	9,0500	1,7848	0,11569
Neutrophils	4,5293	4,5000	1,84400	8,700	3,6320	5,3000	1,3468	0,08978
Neutrophils, %	58,5961	58,7500	35,32000	89,000	53,1400	63,8000	8,1623	0,54295
Platelets	245,9861	245,8500	33,10000	398,000	204,0000	292,6000	62,0118	4,01963
Creatinine	86,2303	83,9000	59,90000	173,000	73,9000	94,1000	16,9088	1,09604
eGFR	94,5004	92,2927	47,00000	192,113	76,0000	107,5556	26,7219	1,73213
Glucose	6,0898	5,4900	2,80000	76,000	5,0000	6,1000	4,8026	0,31463
AST	24,8593	21,0000	10,00000	134,000	17,0000	26,2000	14,9022	0,98049

ALT	30,4563	23,0000	6,00000	199,000	17,0000	34,0000	25,0673	1,64931
Potassium	4,4512	4,4000	3,50000	5,900	4,1000	4,7000	0,4456	0,03097
Intraoperative data								
CPB	0,7750	1,0000	0,00000	1,000	1,0000	1,0000	0,4185	0,02701
CPB time	104,8824	105,0000	0,00000	215,000	80,0000	130,0000	39,8375	2,91321
Cardioplegia	0,2708	0,0000	0,00000	1,000	0,0000	1,0000	0,4453	0,02875
ACC time	63,8254	63,0000	36,00000	127,000	56,0000	70,0000	13,4134	1,68994
CABG	0,8333	1,0000	0,00000	1,000	1,0000	1,0000	0,3735	0,02411
AC - 1	0,5833	1,0000	0,00000	1,000	0,0000	1,0000	0,4940	0,03189
AC - 2	0,0375	0,0000	0,00000	1,000	0,0000	0,0000	0,1904	0,01229
AC - 3	0,0042	0,0000	0,00000	1,000	0,0000	0,0000	0,0645	0,00417
VC - 1	0,3042	0,0000	0,00000	1,000	0,0000	1,0000	0,4610	0,02976
VC - 2	0,2875	0,0000	0,00000	1,000	0,0000	1,0000	0,4535	0,02928
VC - 3	0,1458	0,0000	0,00000	1,000	0,0000	0,0000	0,3537	0,02283
VC - 4	0,0167	0,0000	0,00000	1,000	0,0000	0,0000	0,1283	0,00828
VC - 5+	0,0000	0,0000	0,00000	0,000	0,0000	0,0000	0,0000	0,00000
RCA	0,4042	0,0000	0,00000	1,000	0,0000	1,0000	0,4918	0,03174
AV repair	0,2500	0,0000	0,00000	1,000	0,0000	0,5000	0,4339	0,02801
ICU data								
Cardiotonic support in ICU	0,3417	0,0000	0,00000	1,000	0,0000	1,0000	0,4753	0,03068
Lung ventilation time	10,1719	8,5000	2,00000	34,400	5,8000	14,1000	5,8475	0,38145
Beta-blockers	0,8792	1,0000	0,00000	1,000	1,0000	1,0000	0,3266	0,02108
Statins	0,7083	1,0000	0,00000	1,000	0,0000	1,0000	0,4555	0,02940
ACE inhibitors	0,5667	1,0000	0,00000	1,000	0,0000	1,0000	0,4966	0,03205
Calcium antagonists	0,2333	0,0000	0,00000	1,000	0,0000	0,0000	0,4238	0,02736
Thiazide diuretics	0,1042	0,0000	0,00000	10,000	0,0000	0,0000	0,6858	0,04427
Loop diuretics	0,1083	0,0000	0,00000	1,000	0,0000	0,0000	0,3115	0,02010
Potassium-sparing diuretics	0,3000	0,0000	0,00000	1,000	0,0000	1,0000	0,4592	0,02964
NSAIDs	0,3917	0,0000	0,00000	1,000	0,0000	1,0000	0,4891	0,03157
Acetylsalicylic acid	0,7667	1,0000	0,00000	1,000	1,0000	1,0000	0,4238	0,02736
Other antiaggregant	0,7125	1,0000	0,00000	1,000	0,0000	1,0000	0,4535	0,02928
Nitrates	0,0875	0,0000	0,00000	1,000	0,0000	0,0000	0,2832	0,01828
Antiarrhythmic drugs	0,0458	0,0000	0,00000	1,000	0,0000	0,0000	0,2096	0,01353
Cardiotonic support	0,4542	0,0000	0,00000	1,000	0,0000	1,0000	0,4989	0,03221
Adrenaline	0,0292	0,0000	0,00000	1,000	0,0000	0,0000	0,1686	0,01088
Norepinephrine	0,2000	0,0000	0,00000	1,000	0,0000	0,0000	0,4008	0,02587
Dopamine	0,3625	0,0000	0,00000	1,000	0,0000	1,0000	0,4817	0,03110
Dobutamine	0,0083	0,0000	0,00000	1,000	0,0000	0,0000	0,0911	0,00588
Lmwh/ UFH	0,9292	1,0000	0,00000	1,000	1,0000	1,0000	0,2571	0,01659
Warfarin	0,2500	0,0000	0,00000	1,000	0,0000	0,5000	0,4339	0,02801
Antibiotics	0,9542	1,0000	0,00000	1,000	1,0000	1,0000	0,2096	0,01353
Steroids	0,7250	1,0000	0,00000	1,000	0,0000	1,0000	0,4474	0,02888
3 postoperative day								
LVEF	54,4859	55,0000	28,90000	73,000	52,7000	57,0000	5,4019	0,35619
iESV	22,4921	20,0000	5,60000	67,000	16,7464	24,6575	10,2210	0,78623
iEDV	46,7437	44,4000	20,70000	105,769	39,7790	52,8000	12,8079	0,87553
Pericardial effusion, n	0,1293	0,0000	0,00000	1,000	0,0000	0,0000	0,3363	0,02208

Pericardial effusion, mm	5,4286	5,0000	2,00000	13,000	3,5000	5,0000	3,1790	0,60077
Pleural effusion, n	0,4163	0,0000	0,00000	1,000	0,0000	1,0000	0,4940	0,03236
Pleural effusion, mm	21,0426	18,5000	2,00000	60,000	15,0000	25,0000	10,7653	1,11035
WBC	12,3197	11,7000	5,20000	27,260	9,5260	14,4550	4,0202	0,25951
Neutrophils	34,0151	8,8000	3,43000	4874,000	6,7710	12,2300	342,2674	24,08185
Neutrophils, %	76,5979	77,4900	10,70000	93,000	71,6600	83,8200	10,2607	0,72016
Platelets	202,1271	192,1000	17,10000	404,000	160,0000	244,0000	64,6155	4,21505
Creatinine	77,8910	73,0000	43,70000	196,0000	66,0000	85,8000	20,18079	1,322088
eGFR	106,2284	104,4281	36,20000	223,5028	85,5000	122,4959	30,78887	2,017046
Glucose	8,4328	6,8000	3,90000	99,0000	5,7000	8,4800	9,59515	0,680182
AST	37,2224	31,0000	3,00000	155,0000	22,8000	43,0000	23,96782	1,619595
ALT	26,2618	20,0000	1,30000	238,0000	13,5000	29,0000	26,89688	1,813388
Potassium	6,7943	4,1000	2,50000	464,0000	3,9000	4,4000	34,76508	2,627993
Beta-blockers	0,8750	1,0000	0,00000	1,0000	1,0000	1,0000	0,33141	0,021392
Statins	0,7375	1,0000	0,00000	1,0000	0,0000	1,0000	0,44091	0,028461
ACE inhibitors	0,5625	1,0000	0,00000	1,0000	0,0000	1,0000	0,49712	0,032089
Calcium antagonists	0,2500	0,0000	0,00000	1,0000	0,0000	0,5000	0,43392	0,028009
Thiazide diuretics	0,0708	0,0000	0,00000	1,0000	0,0000	0,0000	0,25708	0,016595
Loop diuretics	0,1583	0,0000	0,00000	1,0000	0,0000	0,0000	0,36582	0,023613
Potassium-sparing diuretics	0,3542	0,0000	0,00000	1,0000	0,0000	1,0000	0,47926	0,030936
NSAIDs	0,4083	0,0000	0,00000	1,0000	0,0000	1,0000	0,49255	0,031794
Acetylsalicylic acid	0,7792	1,0000	0,00000	1,0000	1,0000	1,0000	0,41568	0,026832
Other antiaggregant	0,7000	1,0000	0,00000	1,0000	0,0000	1,0000	0,45922	0,029642
Nitrates	0,0333	0,0000	0,00000	1,0000	0,0000	0,0000	0,17988	0,011611
Antiarrhythmic drugs	0,1083	0,0000	0,00000	1,0000	0,0000	0,0000	0,31145	0,020104
Cardiotonic support	0,2333	0,0000	0,00000	1,0000	0,0000	0,0000	0,42384	0,027359
Adrenaline	0,0167	0,0000	0,00000	1,0000	0,0000	0,0000	0,12829	0,008281
Norepinephrine	0,1000	0,0000	0,00000	1,0000	0,0000	0,0000	0,30063	0,019405
Dopamine	0,1708	0,0000	0,00000	1,0000	0,0000	0,0000	0,37715	0,024345
Dobutamine	0,0083	0,0000	0,00000	1,0000	0,0000	0,0000	0,09110	0,005880
Lmwh/ UFH	0,7958	1,0000	0,00000	1,0000	1,0000	1,0000	0,40393	0,026074
Warfarin	0,2542	0,0000	0,00000	1,0000	0,0000	1,0000	0,43630	0,028163
Antibiotics	0,5000	0,5000	0,00000	1,0000	0,0000	1,0000	0,50104	0,032342
Steroids	0,3667	0,0000	0,00000	1,0000	0,0000	1,0000	0,48290	0,031171
5 postoperative day								
LVEF	55,3275	55,4000	31,60000	73,4000	54,0000	58,0000	5,09454	0,336657
iESV	21,2171	19,6000	9,30000	63,7000	16,4000	23,8000	8,50750	0,652496
iEDV	47,2153	45,0000	22,00000	113,9000	38,5000	53,5000	13,77959	0,953154
Pericardial effusion, n	0,2130	0,0000	0,00000	1,0000	0,0000	0,0000	0,41035	0,027058
Pericardial effusion, mm	5,9574	5,0000	2,00000	40,0000	4,0000	6,0000	5,59875	0,816661
Pleural effusion, n	0,4526	0,0000	0,00000	2,0000	0,0000	1,0000	0,50743	0,033314
Pleural effusion, mm	22,0408	20,0000	3,00000	65,0000	15,0000	28,0000	10,83515	1,094515
WBC	10,3843	9,8810	4,40000	24,4800	8,1250	12,0000	3,30999	0,222152

Neutrophils	6,7645	6,3800	1,90000	15,9000	4,8535	8,0000	2,61500	0,190719
Neutrophils, %	63,4316	62,2500	35,11000	87,9500	57,3000	68,9000	9,69667	0,709091
Platelets	265,1144	265,2000	25,10000	529,8000	212,0000	317,0000	82,06427	5,507793
Creatinine	77,6058	77,0000	39,40000	124,3000	68,1000	85,5000	14,08298	0,976479
eGFR	104,5269	101,5301	52,88889	211,7567	82,9668	121,2323	29,37493	2,036785
Glucose	7,1826	5,9650	4,14000	85,0000	5,3100	7,4000	6,21743	0,460867
AST	38,4200	28,0000	11,00000	627,0000	21,0000	38,0000	61,87316	4,321409
ALT	37,7341	28,0000	3,60000	360,0000	18,0000	43,0000	41,01848	2,864855
Potassium	4,2093	4,2000	1,00000	5,8000	3,9000	4,5000	0,55698	0,043896
Beta-blockers	0,8958	1,0000	0,00000	1,0000	1,0000	1,0000	0,30612	0,019760
Statins	0,7417	1,0000	0,00000	1,0000	0,0000	1,0000	0,43863	0,028314
ACE inhibitors	0,5583	1,0000	0,00000	1,0000	0,0000	1,0000	0,49762	0,032121
Calcium antagonists	0,2542	0,0000	0,00000	1,0000	0,0000	1,0000	0,43630	0,028163
Thiazide diuretics	0,0667	0,0000	0,00000	1,0000	0,0000	0,0000	0,24997	0,016135
Loop diuretics	0,1625	0,0000	0,00000	1,0000	0,0000	0,0000	0,36968	0,023863
Potassium-sparing diuretics	0,3583	0,0000	0,00000	1,0000	0,0000	1,0000	0,48051	0,031017
NSAIDs	0,3500	0,0000	0,00000	1,0000	0,0000	1,0000	0,47797	0,030853
Acetylsalicylic acid	0,8000	1,0000	0,00000	1,0000	1,0000	1,0000	0,40084	0,025874
Other antiaggregant	0,7292	1,0000	0,00000	1,0000	0,0000	1,0000	0,44532	0,028745
Nitrates	0,0292	0,0000	0,00000	1,0000	0,0000	0,0000	0,16863	0,010885
Antiarrhythmic drugs	0,1167	0,0000	0,00000	1,0000	0,0000	0,0000	0,32169	0,020765
Cardiotonic support	0,0542	0,0000	0,00000	1,0000	0,0000	0,0000	0,22682	0,014641
Adrenaline	0,0042	0,0000	0,00000	1,0000	0,0000	0,0000	0,06455	0,004167
Norepinephrine	0,0167	0,0000	0,00000	1,0000	0,0000	0,0000	0,12829	0,008281
Dopamine	0,0375	0,0000	0,00000	1,0000	0,0000	0,0000	0,19038	0,012289
Dobutamine	0,0083	0,0000	0,00000	1,0000	0,0000	0,0000	0,09110	0,005880
Lmwh/ UFH	0,6208	1,0000	0,00000	1,0000	0,0000	1,0000	0,48619	0,031384
Warfarin	0,2625	0,0000	0,00000	1,0000	0,0000	1,0000	0,44091	0,028461
Antibiotics	0,3333	0,0000	0,00000	1,0000	0,0000	1,0000	0,47239	0,030493
Steroids	0,1333	0,0000	0,00000	1,0000	0,0000	0,0000	0,34065	0,021989
Outcomes								
POAF	0,2500	0,0000	0,00000	1,0000	0,0000	0,5000	0,43392	0,028009
POAF day	6,2833	7,0000	1,00000	15,0000	5,0000	7,0000	2,85840	0,184509
Effective management of POAF	0,9667	1,0000	0,00000	1,0000	1,0000	1,0000	0,18102	0,023370
Hospital mortality	0,0000	0,0000	0,00000	0,0000	0,0000	0,0000	0,00000	0,000000
LOS	7,7167	7,0000	5,00000	15,000	6,0000	8,0000	2,0195	0,13036
Respiratory failure	0,0000	0,0000	0,00000	0,0000	0,0000	0,0000	0,00000	0,000000
Stroke	0,0000	0,0000	0,00000	0,0000	0,0000	0,0000	0,00000	0,000000
Bleeding	0,0000	0,0000	0,00000	0,0000	0,0000	0,0000	0,00000	0,000000
VESs	0,0042	0,0000	0,00000	1,0000	0,0000	0,0000	0,06455	0,004167
Infectious complications of postoperative wound	0,0000	0,0000	0,00000	0,0000	0,0000	0,0000	0,00000	0,000000

Arrhythmias, except AF	0,0333	0,0000	0,00000	1,0000	0,0000	0,0000	0,17988	0,011611
Pericardial puncture	0,0042	0,0000	0,00000	1,0000	0,0000	0,0000	0,06455	0,004167
SVESs	0,0208	0,0000	0,00000	1,0000	0,0000	0,0000	0,14312	0,009239
AV-block	0,0125	0,0000	0,00000	1,0000	0,0000	0,0000	0,11133	0,007187
Pacemaker implantation	0,0125	0,0000	0,00000	1,0000	0,0000	0,0000	0,11133	0,007187
Nausea	0,1208	0,0000	0,00000	1,0000	0,0000	0,0000	0,32661	0,021083
Vomiting	0,0333	0,0000	0,00000	1,0000	0,0000	0,0000	0,17988	0,011611
Lack of appetite	0,1792	0,0000	0,00000	1,0000	0,0000	0,0000	0,38429	0,024806
Diarrhea	0,1833	0,0000	0,00000	1,0000	0,0000	0,0000	0,38775	0,025029
Abdominal pain	0,0417	0,0000	0,00000	1,0000	0,0000	0,0000	0,20024	0,012926
Convulsions	0,0375	0,0000	0,00000	1,0000	0,0000	0,0000	0,19038	0,012289
Tingling in the extremities	0,0792	0,0000	0,00000	1,0000	0,0000	0,0000	0,27056	0,017465
Skin rashes	0,0000	0,0000	0,00000	0,0000	0,0000	0,0000	0,00000	0,000000

НАУЧНЫЙ ЦЕНТР СЕРДЕЧНО-СОСУДИСТОЙ ХИРУРГИИ

Placebo n=127								
	Mean	Median	Minimum	Maximum	Lower Quartile	Upper Quartile	Std. Dev.	Std. Error
Age	61,5984	61,0000	43,0000	80,0000	56,0000	67,0000	8,26377	0,733291
Male	0,7638	1,0000	0,0000	1,0000	1,0000	1,0000	0,42644	0,037841
BSA	2,0223	2,0200	1,6000	2,6100	1,8900	2,1300	0,18705	0,016598
Weight	85,6614	85,0000	59,0000	129,0000	76,0000	94,0000	14,11192	1,252231
BMI	29,5114	29,0000	20,0000	46,0700	26,0000	32,3000	4,95420	0,439614
Angina pectoris	0,8819	1,0000	0,0000	1,0000	1,0000	1,0000	0,32402	0,028752
Angina pectoris class III-IV	0,5354	1,0000	0,0000	1,0000	0,0000	1,0000	0,50072	0,044432
Diabetes	0,1890	0,0000	0,0000	1,0000	0,0000	0,0000	0,39304	0,034877
COPD	0,0551	0,0000	0,0000	1,0000	0,0000	0,0000	0,22911	0,020331
Hypertension	0,9370	1,0000	0,0000	1,0000	1,0000	1,0000	0,24391	0,021644
Prior AMI	0,4016	0,0000	0,0000	1,0000	0,0000	1,0000	0,49216	0,043672
Stroke	0,0157	0,0000	0,0000	1,0000	0,0000	0,0000	0,12499	0,011091
Smoking	0,3228	0,0000	0,0000	1,0000	0,0000	1,0000	0,46941	0,041654
Beta-blockers	0,7480	1,0000	0,0000	1,0000	0,0000	1,0000	0,43586	0,038677
ACE inhibitors	0,5669	1,0000	0,0000	1,0000	0,0000	1,0000	0,49746	0,044143
Calcium antagonists	0,3622	0,0000	0,0000	1,0000	0,0000	1,0000	0,48254	0,042819
Thiazide diuretics	0,0551	0,0000	0,0000	1,0000	0,0000	0,0000	0,22911	0,020331
Loop diuretics	0,0787	0,0000	0,0000	1,0000	0,0000	0,0000	0,27040	0,023994
Potassium-sparing diuretics	0,1732	0,0000	0,0000	1,0000	0,0000	0,0000	0,37994	0,033715
NSAIDs	0,0000	0,0000	0,0000	0,0000	0,0000	0,0000	0,00000	0,000000
Acetylsalicylic acid	0,3465	0,0000	0,0000	1,0000	0,0000	1,0000	0,47773	0,042391
Other antiaggregant	0,1732	0,0000	0,0000	1,0000	0,0000	0,0000	0,37994	0,033715
Nitrates	0,1969	0,0000	0,0000	1,0000	0,0000	0,0000	0,39919	0,035423
Statins	0,7953	1,0000	0,0000	1,0000	1,0000	1,0000	0,40510	0,035947
LMWHs/ UFH	0,2283	0,0000	0,0000	1,0000	0,0000	0,0000	0,42143	0,037396
LVEF	59,9220	60,0000	40,0000	77,0000	56,0000	64,0000	6,27975	0,557237
iESD	16,3907	16,4948	1,6667	24,0000	14,9419	18,0000	3,09467	0,292418
iEDD	24,4200	24,1860	14,0000	34,1346	22,7273	26,2376	3,17029	0,298235
iESV	23,3099	21,5122	7,1000	56,8269	18,4000	26,1000	8,51763	0,755818
iEDV	57,4637	55,8416	25,8000	126,7788	46,8000	64,4279	15,26694	1,354722
AV peak gradient	28,2008	9,0000	3,0000	143,0000	6,0000	26,0000	38,11945	3,451173
AV mean gradient	22,0064	5,0000	0,0000	94,0000	3,0000	45,0000	25,92039	2,934907
MR	1,2760	1,5000	0,0000	2,5000	1,0000	1,5000	0,57311	0,051260
AR	0,6807	0,0000	0,0000	3,0000	0,0000	1,0000	0,85055	0,077970
IVS	13,3333	13,0000	9,0000	21,0000	12,0000	15,0000	2,36666	0,213395
LA size	4,9713	4,0000	2,9000	43,0000	3,7000	4,4000	5,95840	0,528722
WBC	7,7388	7,6020	4,1000	12,2000	6,5750	9,0650	1,69069	0,151220
Neutrophils	4,5391	4,7000	1,8440	8,7000	3,7170	5,2700	1,21379	0,110803
Neutrophils, %	58,4017	58,9000	41,0300	82,0000	53,1500	62,9600	7,69502	0,699548
Platelets	248,8720	257,8000	103,0000	373,0000	213,0000	297,8000	59,56150	5,327343
Creatinine	86,3920	84,0000	60,0000	173,0000	74,0000	95,0000	17,70630	1,583700
eGFR	94,6062	89,3883	47,0000	166,8741	75,5041	109,7609	26,41698	2,362806
Glucose	6,3574	5,4000	4,0800	76,0000	4,9200	5,8100	6,57692	0,597902
AST	24,6855	20,0000	11,8000	134,0000	17,0000	26,0000	15,31503	1,375330
ALT	31,5242	24,5000	8,0000	199,0000	18,0000	34,0000	26,48473	2,378399

Potassium	4,4434	4,4000	3,5000	5,9000	4,1000	4,7000	0,43425	0,042178
Intraoperative data								
CPB	0,7874	1,0000	0,0000	1,0000	1,0000	1,0000	0,41077	0,036450
CPB time	107,4600	108,0000	25,0000	190,0000	80,0000	130,5000	37,32641	3,732641
Cardioplegia	0,2598	0,0000	0,0000	1,0000	0,0000	1,0000	0,44028	0,039069
ACC time	62,8710	63,0000	36,0000	95,0000	54,0000	70,0000	13,24825	2,379456
CABG	0,8504	1,0000	0,0000	1,0000	1,0000	1,0000	0,35810	0,031776
AC - 1	0,6063	1,0000	0,0000	1,0000	0,0000	1,0000	0,49050	0,043525
AC - 2	0,0394	0,0000	0,0000	1,0000	0,0000	0,0000	0,19524	0,017325
AC - 3	0,0079	0,0000	0,0000	1,0000	0,0000	0,0000	0,08874	0,007874
VC - 1	0,2677	0,0000	0,0000	1,0000	0,0000	1,0000	0,44452	0,039445
VC - 2	0,2677	0,0000	0,0000	1,0000	0,0000	1,0000	0,44452	0,039445
VC - 3	0,2047	0,0000	0,0000	1,0000	0,0000	0,0000	0,40510	0,035947
VC - 4	0,0079	0,0000	0,0000	1,0000	0,0000	0,0000	0,08874	0,007874
VC - 5+	0,0000	0,0000	0,0000	0,0000	0,0000	0,0000	0,00000	0,000000
RCA	0,4724	0,0000	0,0000	1,0000	0,0000	1,0000	0,50122	0,044476
AV repair	0,2205	0,0000	0,0000	1,0000	0,0000	0,0000	0,41621	0,036932
ICU data								
Cardiotonic support in ICU	0,2677	0,0000	0,0000	1,0000	0,0000	1,0000	0,44452	0,039445
Lung ventilation time	10,3394	8,6000	2,0000	29,2000	5,8000	14,8000	5,96869	0,529636
Beta-blockers	0,8819	1,0000	0,0000	1,0000	1,0000	1,0000	0,32402	0,028752
Statins	0,7087	1,0000	0,0000	1,0000	0,0000	1,0000	0,45618	0,040479
ACE inhibitors	0,5433	1,0000	0,0000	1,0000	0,0000	1,0000	0,50009	0,044376
Calcium antagonists	0,2362	0,0000	0,0000	1,0000	0,0000	0,0000	0,42644	0,037841
Thiazide diuretics	0,1339	0,0000	0,0000	10,0000	0,0000	0,0000	0,91167	0,080898
Loop diuretics	0,1024	0,0000	0,0000	1,0000	0,0000	0,0000	0,30432	0,027004
Potassium-sparing diuretics	0,2441	0,0000	0,0000	1,0000	0,0000	0,0000	0,43125	0,038267
NSAIDs	0,3780	0,0000	0,0000	1,0000	0,0000	1,0000	0,48680	0,043196
Acetylsalicylic acid	0,7402	1,0000	0,0000	1,0000	0,0000	1,0000	0,44028	0,039069
Other antiaggregant	0,7559	1,0000	0,0000	1,0000	1,0000	1,0000	0,43125	0,038267
Nitrates	0,1181	0,0000	0,0000	1,0000	0,0000	0,0000	0,32402	0,028752
Antiarrhythmic drugs	0,0394	0,0000	0,0000	1,0000	0,0000	0,0000	0,19524	0,017325
Cardiotonic support	0,4646	0,0000	0,0000	1,0000	0,0000	1,0000	0,50072	0,044432
Adrenaline	0,0236	0,0000	0,0000	1,0000	0,0000	0,0000	0,15247	0,013530
Norepinephrine	0,1890	0,0000	0,0000	1,0000	0,0000	0,0000	0,39304	0,034877
Dopamine	0,3780	0,0000	0,0000	1,0000	0,0000	1,0000	0,48680	0,043196
Dobutamine	0,0000	0,0000	0,0000	0,0000	0,0000	0,0000	0,00000	0,000000
Lmwh/ UFH	0,9213	1,0000	0,0000	1,0000	1,0000	1,0000	0,27040	0,023994
Warfarin	0,2283	0,0000	0,0000	1,0000	0,0000	0,0000	0,42143	0,037396
Antibiotics	0,9449	1,0000	0,0000	1,0000	1,0000	1,0000	0,22911	0,020331
Steroids	0,7717	1,0000	0,0000	1,0000	1,0000	1,0000	0,42143	0,037396
3 postoperative day								
LVEF	54,9429	55,0000	39,0000	73,0000	53,0000	57,0000	4,84602	0,431718
iESV	21,8383	20,1275	5,6000	53,8462	17,3099	23,8191	8,28994	0,855042
iEDV	45,6399	44,4000	20,7000	105,7692	39,4000	51,2000	11,38245	1,066064
Pericardial effusion, n	0,1587	0,0000	0,0000	1,0000	0,0000	0,0000	0,36688	0,032685

Pericardial effusion, mm	6,2500	5,0000	2,0000	13,0000	5,0000	9,0000	3,38534	0,756985
Pleural effusion, n	0,4048	0,0000	0,0000	1,0000	0,0000	1,0000	0,49281	0,043903
Pleural effusion, mm	22,4898	20,0000	2,0000	47,0000	15,0000	26,0000	10,50421	1,500601
WBC	12,5885	12,0000	5,2000	27,2600	9,6000	14,6000	4,19723	0,372444
Neutrophils	10,3676	8,9300	4,2000	49,0000	6,7890	12,7000	5,61350	0,555820
Neutrophils, %	76,7157	78,9800	10,7000	92,1300	72,0000	82,8300	10,08423	0,993628
Platelets	204,6919	199,0000	17,1000	404,0000	162,0000	254,0000	67,74974	6,108788
Creatinine	80,3750	75,0000	46,70000	196,0000	66,7000	88,5000	22,24657	1,997801
eGFR	103,7676	100,0465	36,20000	223,5028	83,7336	124,1149	31,99815	2,873519
Glucose	7,5327	6,6200	4,15000	28,0000	5,7000	8,3000	3,28504	0,323685
AST	37,9042	31,0000	3,00000	155,0000	21,0000	40,0000	27,53081	2,534416
ALT	27,8588	21,0000	1,30000	238,0000	13,0000	29,0000	33,19785	3,043242
Potassium	9,2707	4,2000	2,50000	464,0000	3,9000	4,5000	47,93446	4,997513
Beta-blockers	0,8898	1,0000	0,00000	1,0000	1,0000	1,0000	0,31442	0,027901
Statins	0,7323	1,0000	0,00000	1,0000	0,0000	1,0000	0,44452	0,039445
ACE inhibitors	0,5512	1,0000	0,00000	1,0000	0,0000	1,0000	0,49934	0,044310
Calcium antagonists	0,2362	0,0000	0,00000	1,0000	0,0000	0,0000	0,42644	0,037841
Thiazide diuretics	0,0630	0,0000	0,00000	1,0000	0,0000	0,0000	0,24391	0,021644
Loop diuretics	0,1654	0,0000	0,00000	1,0000	0,0000	0,0000	0,37297	0,033096
Potassium-sparing diuretics	0,3071	0,0000	0,00000	1,0000	0,0000	1,0000	0,46311	0,041095
NSAIDs	0,3858	0,0000	0,00000	1,0000	0,0000	1,0000	0,48872	0,043367
Acetylsalicylic acid	0,7795	1,0000	0,00000	1,0000	1,0000	1,0000	0,41621	0,036932
Other antiaggregant	0,7402	1,0000	0,00000	1,0000	0,0000	1,0000	0,44028	0,039069
Nitrates	0,0551	0,0000	0,00000	1,0000	0,0000	0,0000	0,22911	0,020331
Antiarrhythmic drugs	0,1102	0,0000	0,00000	1,0000	0,0000	0,0000	0,31442	0,027901
Cardiotonic support	0,2126	0,0000	0,00000	1,0000	0,0000	0,0000	0,41077	0,036450
Adrenaline	0,0236	0,0000	0,00000	1,0000	0,0000	0,0000	0,15247	0,013530
Norepinephrine	0,0787	0,0000	0,00000	1,0000	0,0000	0,0000	0,27040	0,023994
Dopamine	0,1496	0,0000	0,00000	1,0000	0,0000	0,0000	0,35810	0,031776
Dobutamine	0,0000	0,0000	0,00000	0,0000	0,0000	0,0000	0,00000	0,000000
Lmwh/ UFH	0,7874	1,0000	0,00000	1,0000	1,0000	1,0000	0,41077	0,036450
Warfarin	0,2362	0,0000	0,00000	1,0000	0,0000	0,0000	0,42644	0,037841
Antibiotics	0,5433	1,0000	0,00000	1,0000	0,0000	1,0000	0,50009	0,044376
Steroids	0,4331	0,0000	0,00000	1,0000	0,0000	1,0000	0,49746	0,044143
5 postoperative day								
LVEF	55,8467	55,2000	43,20000	73,4000	54,0000	58,0000	4,61797	0,418091
iESV	20,7472	19,6000	9,70000	57,2000	16,6000	23,8000	6,70978	0,711235
iEDV	46,3694	45,0000	22,00000	113,9000	38,5000	53,9000	11,56333	1,097543
Pericardial effusion, n	0,2213	0,0000	0,00000	1,0000	0,0000	0,0000	0,41684	0,037739
Pericardial effusion, mm	5,6800	5,0000	4,00000	15,0000	5,0000	6,0000	2,23084	0,446169
Pleural effusion, n	0,4715	0,0000	0,00000	1,0000	0,0000	1,0000	0,50123	0,045195
Pleural effusion, mm	24,2222	20,0000	10,00000	65,0000	15,0000	32,0000	11,72108	1,595037

WBC	11,0789	10,9300	4,40000	24,4800	8,4000	13,2000	3,68177	0,349458
Neutrophils	7,2641	6,8300	1,90000	15,9000	5,0000	9,4000	2,85973	0,288876
Neutrophils, %	63,3685	62,9800	35,11000	87,0500	57,0000	68,3900	10,18030	1,028366
Platelets	267,1928	262,3000	25,10000	445,0000	217,6000	329,2000	81,47362	7,733134
Creatinine	78,2373	77,2000	58,70000	124,3000	68,2000	86,0000	13,28917	1,315824
eGFR	104,5843	100,0776	59,53201	211,7567	82,0000	127,0646	30,67446	3,037224
Glucose	7,4896	5,9200	4,20000	85,0000	5,3350	7,3000	8,26083	0,843118
AST	45,0850	29,0000	14,00000	627,0000	20,0000	38,0000	86,03496	8,603496
ALT	42,9560	27,0000	3,60000	360,0000	17,3500	46,0000	54,69482	5,469482
Potassium	4,2952	4,3000	1,00000	5,8000	4,0000	4,6000	0,62350	0,068438
Beta-blockers	0,9055	1,0000	0,00000	1,0000	1,0000	1,0000	0,29367	0,026059
Statins	0,7402	1,0000	0,00000	1,0000	0,0000	1,0000	0,44028	0,039069
ACE inhibitors	0,5354	1,0000	0,00000	1,0000	0,0000	1,0000	0,50072	0,044432
Calcium antagonists	0,2598	0,0000	0,00000	1,0000	0,0000	1,0000	0,44028	0,039069
Thiazide diuretics	0,0551	0,0000	0,00000	1,0000	0,0000	0,0000	0,22911	0,020331
Loop diuretics	0,1496	0,0000	0,00000	1,0000	0,0000	0,0000	0,35810	0,031776
Potassium-sparing diuretics	0,2913	0,0000	0,00000	1,0000	0,0000	1,0000	0,45618	0,040479
NSAIDs	0,3150	0,0000	0,00000	1,0000	0,0000	1,0000	0,46634	0,041381
Acetylsalicylic acid	0,7795	1,0000	0,00000	1,0000	1,0000	1,0000	0,41621	0,036932
Other antiaggregant	0,7638	1,0000	0,00000	1,0000	1,0000	1,0000	0,42644	0,037841
Nitrates	0,0551	0,0000	0,00000	1,0000	0,0000	0,0000	0,22911	0,020331
Antiarrhythmic drugs	0,1496	0,0000	0,00000	1,0000	0,0000	0,0000	0,35810	0,031776
Cardiotonic support	0,0236	0,0000	0,00000	1,0000	0,0000	0,0000	0,15247	0,013530
Adrenaline	0,0000	0,0000	0,00000	0,0000	0,0000	0,0000	0,00000	0,000000
Norepinephrine	0,0079	0,0000	0,00000	1,0000	0,0000	0,0000	0,08874	0,007874
Dopamine	0,0157	0,0000	0,00000	1,0000	0,0000	0,0000	0,12499	0,011091
Dobutamine	0,0000	0,0000	0,00000	0,0000	0,0000	0,0000	0,00000	0,000000
Lmwh/ UFH	0,5906	1,0000	0,00000	1,0000	0,0000	1,0000	0,49368	0,043807
Warfarin	0,2520	0,0000	0,00000	1,0000	0,0000	1,0000	0,43586	0,038677
Antibiotics	0,3701	0,0000	0,00000	1,0000	0,0000	1,0000	0,48474	0,043014
Steroids	0,1102	0,0000	0,00000	1,0000	0,0000	0,0000	0,31442	0,027901
Outcomes								
POAF	0,3071	0,0000	0,00000	1,0000	0,0000	1,0000	0,46311	0,041095
POAF day	5,9764	6,0000	1,00000	15,0000	3,0000	7,0000	3,03803	0,269581
Effective management of POAF	0,9487	1,0000	0,00000	1,0000	1,0000	1,0000	0,22346	0,035782
Hospital mortality	0,0000	0,0000	0,00000	0,0000	0,0000	0,0000	0,00000	0,000000
LOS	7,8031	7,0000	5,0000	15,0000	6,0000	9,0000	2,23266	0,198116
Respiratory failure	0,0000	0,0000	0,00000	0,0000	0,0000	0,0000	0,00000	0,000000
Stroke	0,0000	0,0000	0,00000	0,0000	0,0000	0,0000	0,00000	0,000000
Bleeding	0,0000	0,0000	0,00000	0,0000	0,0000	0,0000	0,00000	0,000000
VESs	0,0000	0,0000	0,00000	0,0000	0,0000	0,0000	0,00000	0,000000
Infectious complications of postoperative wound	0,0000	0,0000	0,00000	0,0000	0,0000	0,0000	0,00000	0,000000

Arrhythmias, except AF	0,0315	0,0000	0,00000	1,0000	0,0000	0,0000	0,17535	0,015559
Pericardial puncture	0,0079	0,0000	0,00000	1,0000	0,0000	0,0000	0,08874	0,007874
SVESs	0,0315	0,0000	0,00000	1,0000	0,0000	0,0000	0,17535	0,015559
AV-block	0,0000	0,0000	0,00000	0,0000	0,0000	0,0000	0,00000	0,000000
Pacemaker implantation	0,0000	0,0000	0,00000	0,0000	0,0000	0,0000	0,00000	0,000000
Nausea	0,1181	0,0000	0,00000	1,0000	0,0000	0,0000	0,32402	0,028752
Vomiting	0,0472	0,0000	0,00000	1,0000	0,0000	0,0000	0,21300	0,018901
Lack of appetite	0,1890	0,0000	0,00000	1,0000	0,0000	0,0000	0,39304	0,034877
Diarrhea	0,1181	0,0000	0,00000	1,0000	0,0000	0,0000	0,32402	0,028752
Abdominal pain	0,0157	0,0000	0,00000	1,0000	0,0000	0,0000	0,12499	0,011091
Convulsions	0,0551	0,0000	0,00000	1,0000	0,0000	0,0000	0,22911	0,020331
Tingling in the extremities	0,0787	0,0000	0,00000	1,0000	0,0000	0,0000	0,27040	0,023994
Skin rashes	0,0000	0,0000	0,00000	0,0000	0,0000	0,0000	0,00000	0,000000

НАУЧНЫЙ ЦЕНТР СЕРДЕЧНО-СОСУДИСТОЙ ХИРУРГИИ

Colchicine n=113								
	Mean	Median	Minimum	Maximum	Lower Quartile	Upper Quartile	Std. Dev.	Std. Error
Age	61,0354	62,0000	42,00000	76,000	55,0000	67,0000	8,3804	0,78836
Male	0,7345	1,0000	0,00000	1,000	0,0000	1,0000	0,4436	0,04173
BSA	2,0209	2,0100	1,53000	2,470	1,8800	2,1800	0,2145	0,02018
Weight	85,2478	84,0000	54,00000	120,000	74,0000	94,0000	15,2939	1,43873
BMI	29,1835	28,7300	21,27000	39,180	25,9600	31,9600	4,1890	0,39407
Angina pectoris	0,9115	1,0000	0,00000	1,000	1,0000	1,0000	0,2853	0,02684
Angina pectoris class III-IV	0,5221	1,0000	0,00000	1,000	0,0000	1,0000	0,5017	0,04720
Diabetes	0,2478	0,0000	0,00000	1,000	0,0000	0,0000	0,4337	0,04079
COPD	0,0619	0,0000	0,00000	1,000	0,0000	0,0000	0,2421	0,02278
Hypertension	0,8850	1,0000	0,00000	1,000	1,0000	1,0000	0,3205	0,03015
Prior AMI	0,4071	0,0000	0,00000	1,000	0,0000	1,0000	0,4935	0,04642
Stroke	0,0177	0,0000	0,00000	1,000	0,0000	0,0000	0,1324	0,01246
Smoking	0,2212	0,0000	0,00000	1,000	0,0000	0,0000	0,4169	0,03922
Beta-blockers	0,7434	1,0000	0,00000	1,000	0,0000	1,0000	0,4387	0,04127
ACE inhibitors	0,6106	1,0000	0,00000	1,000	0,0000	1,0000	0,4898	0,04607
Calcium antagonists	0,3274	0,0000	0,00000	1,000	0,0000	1,0000	0,4714	0,04434
Thiazide diuretics	0,1239	0,0000	0,00000	1,000	0,0000	0,0000	0,3309	0,03113
Loop diuretics	0,1150	0,0000	0,00000	1,000	0,0000	0,0000	0,3205	0,03015
Potassium-sparing diuretics	0,2124	0,0000	0,00000	1,000	0,0000	0,0000	0,4108	0,03865
NSAIDs	0,0000	0,0000	0,00000	0,000	0,0000	0,0000	0,0000	0,00000
Acetylsalicylic acid	0,3186	0,0000	0,00000	1,000	0,0000	1,0000	0,4680	0,04403
Other antiaggregant	0,1947	0,0000	0,00000	1,000	0,0000	0,0000	0,3977	0,03741
Nitrates	0,2566	0,0000	0,00000	1,000	0,0000	1,0000	0,4387	0,04127
Statins	0,6814	1,0000	0,00000	1,000	0,0000	1,0000	0,4680	0,04403
LMWHs/ UFH	0,2389	0,0000	0,00000	1,000	0,0000	0,0000	0,4283	0,04029
LVEF	58,8274	60,0000	36,20000	72,800	56,0000	64,0000	6,6570	0,62624
iESD	16,9284	16,5000	11,80000	28,800	15,3465	18,1000	2,9430	0,29578
iEDD	24,7509	24,5192	17,30000	35,000	22,7723	26,5700	3,3854	0,34024
iESV	24,3914	22,6519	11,04972	69,900	18,7624	26,7648	9,5404	0,90149
iEDV	58,6904	56,3000	28,20000	109,600	48,6000	66,7000	15,6635	1,47350
AV peak gradient	29,1745	8,0000	3,00000	134,000	6,0000	45,0000	36,2104	3,45253
AV mean gradient	19,6250	6,0000	0,00000	74,000	3,2500	41,0000	22,2292	2,42541
MR	1,3438	1,5000	0,00000	2,000	1,0000	1,5000	0,4747	0,04486
AR	0,7336	1,0000	0,00000	3,000	0,0000	1,0000	0,8420	0,08140
IVS	13,2920	13,0000	7,00000	22,000	12,0000	15,0000	2,7829	0,26179
LA size	4,0721	4,0000	2,90000	5,300	3,8000	4,4000	0,4679	0,04441
WBC	7,6058	7,4590	3,69000	12,000	6,4000	8,9000	1,8886	0,17766
Neutrophils	4,5181	4,4320	1,90700	8,600	3,4000	5,4000	1,4901	0,14542
Neutrophils, %	58,8202	58,0000	35,32000	89,000	53,0000	65,0000	8,7017	0,84919
Platelets	242,7938	236,0000	33,10000	398,000	197,0000	279,0000	64,7305	6,08933
Creatinine	86,0513	83,8000	59,90000	133,000	72,9000	93,6000	16,0573	1,51054
eGFR	94,3834	92,6572	51,46199	192,113	77,0000	106,4772	27,1726	2,55619
Glucose	5,8007	5,6000	2,80000	9,800	5,0550	6,4000	1,1328	0,10704
AST	25,0607	22,0000	10,00000	123,000	17,0000	27,0000	14,4782	1,39966
ALT	29,2187	23,0000	6,00000	191,000	16,0000	33,0000	23,3802	2,26025

Potassium	4,4594	4,4000	3,50000	5,500	4,1000	4,8000	0,4592	0,04569
Intraoperative data								
CPB	0,7611	1,0000	0,00000	1,000	1,0000	1,0000	0,4283	0,04029
CPB time	101,9195	105,0000	0,00000	215,000	75,0000	130,0000	42,5668	4,56364
Cardioplegia	0,2832	0,0000	0,00000	1,000	0,0000	1,0000	0,4526	0,04257
ACC time	64,7500	62,5000	50,00000	127,000	57,0000	66,5000	13,7184	2,42509
CABG	0,8142	1,0000	0,00000	1,000	1,0000	1,0000	0,3907	0,03675
AC - 1	0,5575	1,0000	0,00000	1,000	0,0000	1,0000	0,4989	0,04693
AC - 2	0,0354	0,0000	0,00000	1,000	0,0000	0,0000	0,1856	0,01746
AC - 3	0,0000	0,0000	0,00000	0,000	0,0000	0,0000	0,0000	0,00000
VC - 1	0,3451	0,0000	0,00000	1,000	0,0000	1,0000	0,4775	0,04492
VC - 2	0,3097	0,0000	0,00000	1,000	0,0000	1,0000	0,4644	0,04369
VC - 3	0,0796	0,0000	0,00000	1,000	0,0000	0,0000	0,2720	0,02558
VC - 4	0,0265	0,0000	0,00000	1,000	0,0000	0,0000	0,1615	0,01519
VC - 5+	0,0000	0,0000	0,00000	0,000	0,0000	0,0000	0,0000	0,00000
RCA	0,3274	0,0000	0,00000	1,000	0,0000	1,0000	0,4714	0,04434
AV repair	0,2832	0,0000	0,00000	1,000	0,0000	1,0000	0,4526	0,04257
ICU data								
Cardiotonic support in ICU	0,4248	0,0000	0,00000	1,000	0,0000	1,0000	0,4965	0,04671
Lung ventilation time	9,9750	8,2500	2,50000	34,400	5,8000	13,6000	5,7230	0,55070
Beta-blockers	0,8761	1,0000	0,00000	1,000	1,0000	1,0000	0,3309	0,03113
Statins	0,7080	1,0000	0,00000	1,000	0,0000	1,0000	0,4567	0,04296
ACE inhibitors	0,5929	1,0000	0,00000	1,000	0,0000	1,0000	0,4935	0,04642
Calcium antagonists	0,2301	0,0000	0,00000	1,000	0,0000	0,0000	0,4228	0,03977
Thiazide diuretics	0,0708	0,0000	0,00000	1,000	0,0000	0,0000	0,2576	0,02424
Loop diuretics	0,1150	0,0000	0,00000	1,000	0,0000	0,0000	0,3205	0,03015
Potassium-sparing diuretics	0,3628	0,0000	0,00000	1,000	0,0000	1,0000	0,4830	0,04543
NSAIDs	0,4071	0,0000	0,00000	1,000	0,0000	1,0000	0,4935	0,04642
Acetylsalicylic acid	0,7965	1,0000	0,00000	1,000	1,0000	1,0000	0,4044	0,03805
Other antiaggregant	0,6637	1,0000	0,00000	1,000	0,0000	1,0000	0,4745	0,04464
Nitrates	0,0531	0,0000	0,00000	1,000	0,0000	0,0000	0,2252	0,02119
Antiarrhythmic drugs	0,0531	0,0000	0,00000	1,000	0,0000	0,0000	0,2252	0,02119
Cardiotonic support	0,4425	0,0000	0,00000	1,000	0,0000	1,0000	0,4989	0,04693
Adrenaline	0,0354	0,0000	0,00000	1,000	0,0000	0,0000	0,1856	0,01746
Norepinephrine	0,2124	0,0000	0,00000	1,000	0,0000	0,0000	0,4108	0,03865
Dopamine	0,3451	0,0000	0,00000	1,000	0,0000	1,0000	0,4775	0,04492
Dobutamine	0,0177	0,0000	0,00000	1,000	0,0000	0,0000	0,1324	0,01246
Lmwh/ UFH	0,9381	1,0000	0,00000	1,000	1,0000	1,0000	0,2421	0,02278
Warfarin	0,2743	0,0000	0,00000	1,000	0,0000	1,0000	0,4482	0,04216
Antibiotics	0,9646	1,0000	0,00000	1,000	1,0000	1,0000	0,1856	0,01746
Steroids	0,6726	1,0000	0,00000	1,000	0,0000	1,0000	0,4714	0,04434
3 postoperative day								
LVEF	53,9322	55,0000	28,90000	68,200	51,9250	56,8000	5,9843	0,58681
iESV	23,3117	19,8000	7,21649	67,000	16,2000	25,2000	12,2302	1,41223
iEDV	48,0020	44,3500	22,68041	104,500	39,8895	54,3689	14,2147	1,42147
Pericardial effusion, n	0,0943	0,0000	0,00000	1,000	0,0000	0,0000	0,2937	0,02853

Pericardial effusion, mm	3,3750	3,5000	2,00000	5,000	2,5000	4,0000	1,0607	0,37500
Pleural effusion, n	0,4299	0,0000	0,00000	1,000	0,0000	1,0000	0,4974	0,04808
Pleural effusion, mm	19,4667	18,0000	2,00000	60,000	12,0000	20,0000	10,9412	1,63101
WBC	12,0175	11,4000	5,69000	22,000	9,5000	14,2000	3,8074	0,35817
Neutrophils	58,1356	8,7875	3,43000	4874,000	6,7355	12,0000	486,4646	48,64646
Neutrophils, %	76,4766	77,3300	21,30000	93,000	71,3800	83,9500	10,4887	1,04887
Platelets	199,3105	189,0000	76,00000	383,000	160,0000	237,0000	61,1678	5,77982
Creatinine	75,0651	71,9000	43,70000	150,0000	64,9000	81,9000	17,20678	1,648110
eGFR	109,0278	107,0000	57,53846	200,5882	90,1677	118,4615	29,24718	2,801371
Glucose	9,3985	7,0200	3,90000	99,0000	5,6950	8,6100	13,35961	1,363510
AST	36,4257	31,0000	12,00000	107,0000	24,0000	45,0000	19,08741	1,899268
ALT	24,3802	20,0000	6,00000	94,0000	14,0000	30,0000	16,65093	1,656829
Potassium	4,0494	4,1000	3,00000	5,4000	3,8000	4,3000	0,45462	0,049901
Beta-blockers	0,8584	1,0000	0,00000	1,0000	1,0000	1,0000	0,35019	0,032943
Statins	0,7434	1,0000	0,00000	1,0000	0,0000	1,0000	0,43872	0,041272
ACE inhibitors	0,5752	1,0000	0,00000	1,0000	0,0000	1,0000	0,49651	0,046708
Calcium antagonists	0,2655	0,0000	0,00000	1,0000	0,0000	1,0000	0,44356	0,041727
Thiazide diuretics	0,0796	0,0000	0,00000	1,0000	0,0000	0,0000	0,27195	0,025583
Loop diuretics	0,1504	0,0000	0,00000	1,0000	0,0000	0,0000	0,35910	0,033781
Potassium-sparing diuretics	0,4071	0,0000	0,00000	1,0000	0,0000	1,0000	0,49348	0,046423
NSAIDs	0,4336	0,0000	0,00000	1,0000	0,0000	1,0000	0,49778	0,046827
Acetylsalicylic acid	0,7788	1,0000	0,00000	1,0000	1,0000	1,0000	0,41693	0,039221
Other antiaggregant	0,6549	1,0000	0,00000	1,0000	0,0000	1,0000	0,47753	0,044922
Nitrates	0,0088	0,0000	0,00000	1,0000	0,0000	0,0000	0,09407	0,008850
Antiarrhythmic drugs	0,1062	0,0000	0,00000	1,0000	0,0000	0,0000	0,30946	0,029111
Cardiotonic support	0,2566	0,0000	0,00000	1,0000	0,0000	1,0000	0,43872	0,041272
Adrenaline	0,0088	0,0000	0,00000	1,0000	0,0000	0,0000	0,09407	0,008850
Norepinephrine	0,1239	0,0000	0,00000	1,0000	0,0000	0,0000	0,33093	0,031131
Dopamine	0,1947	0,0000	0,00000	1,0000	0,0000	0,0000	0,39773	0,037415
Dobutamine	0,0177	0,0000	0,00000	1,0000	0,0000	0,0000	0,13244	0,012459
Lmwh/ UFH	0,8053	1,0000	0,00000	1,0000	1,0000	1,0000	0,39773	0,037415
Warfarin	0,2743	0,0000	0,00000	1,0000	0,0000	1,0000	0,44817	0,042160
Antibiotics	0,4513	0,0000	0,00000	1,0000	0,0000	1,0000	0,49984	0,047021
Steroids	0,2920	0,0000	0,00000	1,0000	0,0000	1,0000	0,45672	0,042965
5 postoperative day								
LVEF	54,7355	55,6000	31,60000	66,3000	53,0000	58,0000	5,55148	0,536682
iESV	21,7333	19,7000	9,30000	63,7000	16,2000	23,4000	10,14194	1,126883
iEDV	48,1735	45,2500	22,20000	106,0000	38,3000	53,3000	15,93053	1,609226
Pericardial effusion, n	0,2037	0,0000	0,00000	1,0000	0,0000	0,0000	0,40463	0,038935
Pericardial effusion, mm	6,2727	5,0000	2,00000	40,0000	3,0000	6,0000	7,92333	1,689260
Pleural effusion, n	0,4312	0,0000	0,00000	2,0000	0,0000	1,0000	0,51581	0,049405
Pleural effusion, mm	19,3636	19,5000	3,00000	40,0000	10,0000	26,0000	9,06589	1,366734

WBC	9,6896	9,3000	4,60000	21,3000	8,0000	11,1000	2,73540	0,259632
Neutrophils	6,2205	5,9610	2,40000	12,4600	4,7000	7,5260	2,20923	0,232873
Neutrophils, %	63,5010	62,0000	40,10000	87,9500	58,6000	68,9000	9,19176	0,974325
Platelets	263,0360	265,4000	33,30000	529,8000	211,0000	314,1000	82,96773	7,874948
Creatinine	76,9981	75,3500	39,40000	120,7000	68,0000	83,1000	14,84451	1,441827
eGFR	104,4717	102,7991	52,88889	194,3898	85,3373	119,2798	28,21409	2,740395
Glucose	6,8399	6,3650	4,14000	15,1600	5,2500	7,4000	2,41257	0,260154
AST	32,0724	28,0000	11,00000	160,5000	22,0000	34,1000	19,48826	1,901859
ALT	32,7610	28,0000	6,00000	138,0000	20,0000	42,0000	20,03569	1,955283
Potassium	4,1179	4,1500	3,00000	5,3000	3,7000	4,4000	0,46284	0,052406
Beta-blockers	0,8850	1,0000	0,00000	1,0000	1,0000	1,0000	0,32050	0,030150
Statins	0,7434	1,0000	0,00000	1,0000	0,0000	1,0000	0,43872	0,041272
ACE inhibitors	0,5841	1,0000	0,00000	1,0000	0,0000	1,0000	0,49508	0,046573
Calcium antagonists	0,2478	0,0000	0,00000	1,0000	0,0000	0,0000	0,43365	0,040794
Thiazide diuretics	0,0796	0,0000	0,00000	1,0000	0,0000	0,0000	0,27195	0,025583
Loop diuretics	0,1770	0,0000	0,00000	1,0000	0,0000	0,0000	0,38336	0,036064
Potassium-sparing diuretics	0,4336	0,0000	0,00000	1,0000	0,0000	1,0000	0,49778	0,046827
NSAIDs	0,3894	0,0000	0,00000	1,0000	0,0000	1,0000	0,48978	0,046075
Acetylsalicylic acid	0,8230	1,0000	0,00000	1,0000	1,0000	1,0000	0,38336	0,036064
Other antiaggregant	0,6903	1,0000	0,00000	1,0000	0,0000	1,0000	0,46444	0,043691
Nitrates	0,0000	0,0000	0,00000	0,0000	0,0000	0,0000	0,00000	0,000000
Antiarrhythmic drugs	0,0796	0,0000	0,00000	1,0000	0,0000	0,0000	0,27195	0,025583
Cardiotonic support	0,0885	0,0000	0,00000	1,0000	0,0000	0,0000	0,28528	0,026837
Adrenaline	0,0088	0,0000	0,00000	1,0000	0,0000	0,0000	0,09407	0,008850
Norepinephrine	0,0265	0,0000	0,00000	1,0000	0,0000	0,0000	0,16148	0,015190
Dopamine	0,0619	0,0000	0,00000	1,0000	0,0000	0,0000	0,24213	0,022778
Dobutamine	0,0177	0,0000	0,00000	1,0000	0,0000	0,0000	0,13244	0,012459
Lmwh/ UFH	0,6549	1,0000	0,00000	1,0000	0,0000	1,0000	0,47753	0,044922
Warfarin	0,2743	0,0000	0,00000	1,0000	0,0000	1,0000	0,44817	0,042160
Antibiotics	0,2920	0,0000	0,00000	1,0000	0,0000	1,0000	0,45672	0,042965
Steroids	0,1593	0,0000	0,00000	1,0000	0,0000	0,0000	0,36758	0,034579
Outcomes								
POAF	0,1858	0,0000	0,00000	1,0000	0,0000	0,0000	0,39071	0,036755
POAF day	6,6283	7,0000	1,00000	15,0000	6,0000	7,0000	2,61245	0,245759
Effective management of POAF	1,0000	1,0000	1,00000	1,0000	1,0000	1,0000	0,00000	0,000000
Hospital mortality	0,0000	0,0000	0,00000	0,0000	0,0000	0,0000	0,00000	0,000000
LOS	7,6071	7,0000	5,00000	15,000	7,0000	8,0000	1,7571	0,16603
Respiratory failure	0,0000	0,0000	0,00000	0,0000	0,0000	0,0000	0,00000	0,000000
Stroke	0,0000	0,0000	0,00000	0,0000	0,0000	0,0000	0,00000	0,000000
Bleeding	0,0000	0,0000	0,00000	0,0000	0,0000	0,0000	0,00000	0,000000
VESs	0,0088	0,0000	0,00000	1,0000	0,0000	0,0000	0,09407	0,008850
Infectious complications of postoperative wound	0,0000	0,0000	0,00000	0,0000	0,0000	0,0000	0,00000	0,000000

Arrhythmias, except AF	0,0354	0,0000	0,00000	1,0000	0,0000	0,0000	0,18561	0,017460
Pericardial puncture	0,0000	0,0000	0,00000	0,0000	0,0000	0,0000	0,00000	0,000000
SVESs	0,0088	0,0000	0,00000	1,0000	0,0000	0,0000	0,09407	0,008850
AV-block	0,0265	0,0000	0,00000	1,0000	0,0000	0,0000	0,16148	0,015190
Pacemaker implantation	0,0265	0,0000	0,00000	1,0000	0,0000	0,0000	0,16148	0,015190
Nausea	0,1239	0,0000	0,00000	1,0000	0,0000	0,0000	0,33093	0,031131
Vomiting	0,0177	0,0000	0,00000	1,0000	0,0000	0,0000	0,13244	0,012459
Lack of appetite	0,1681	0,0000	0,00000	1,0000	0,0000	0,0000	0,37566	0,035339
Diarrhea	0,2566	0,0000	0,00000	1,0000	0,0000	1,0000	0,43872	0,041272
Abdominal pain	0,0708	0,0000	0,00000	1,0000	0,0000	0,0000	0,25763	0,024236
Convulsions	0,0177	0,0000	0,00000	1,0000	0,0000	0,0000	0,13244	0,012459
Tingling in the extremities	0,0796	0,0000	0,00000	1,0000	0,0000	0,0000	0,27195	0,025583
Skin rashes	0,0000	0,0000	0,00000	0,0000	0,0000	0,0000	0,00000	0,000000

НАУЧНЫЙ ЦЕНТР СЕРДЕЧНО-СОСУДИСТОЙ ХИРУРГИИ
